# Associations of Dietary Patterns and Micronutrients With Major Adverse Cardiovascular Events and Mortality Among Populations With Cardiovascular‐Kidney‐Metabolic Syndrome Stages 0–3: Results From Two Prospective Cohorts

**DOI:** 10.1002/fsn3.72082

**Published:** 2026-07-02

**Authors:** Yingqi Hou, Kaixin Yuan, Yadan Xu, Wen Gu, Tian Wang, Tangyi Su, Yangchen Lhamu, Yanqiu Huang, Shuli Li, Jinjin Liu, Honglin Liu, Wentao Shi

**Affiliations:** ^1^ Clinical Research Unit, Shanghai Ninth People's Hospital Shanghai Jiao Tong University School of Medicine Shanghai China; ^2^ Department of Clinical Laboratory Zhongshan Second People's Hospital Zhongshan China; ^3^ Department of Laboratory Medicine Affiliated Hospital of Changchun University of Chinese Medicine Changchun China; ^4^ Department of Nutrition Shanghai Jiao Tong University School of Medicine Shanghai China; ^5^ School of Public Health Shanghai Jiao Tong University School of Medicine Shanghai China; ^6^ Shanghai Jing'an District Zhabei Center Hospital Shanghai China; ^7^ Department of Clinical Laboratory The Third People's Hospital of Liupanshui Liupanshui Guizhou China

**Keywords:** cardiovascular‐kidney‐metabolic syndrome, inflammatory diets, MACE, micronutrients, NHANES, plant‐based diets, UK Biobank

## Abstract

Following the recent proposal of cardiovascular‐kidney‐metabolic (CKM) syndrome by the American Heart Association (AHA), it remains uncertain whether plant‐based diets, inflammatory diets, or micronutrient intake are associated with major adverse cardiovascular events (MACE) and mortality in patients with CKM syndrome stages 0–3. The cohort study data were from the National Health and Nutrition Examination Survey (NHANES) 2005–2018 and the UK Biobank (UKB). We calculated scores of plant‐based diets, inflammatory diet in NHANES (dietary inflammatory index (DII) and alternative Mediterranean diet (AMED)), and UKB (AMED and Healthful Plant‐Based Diet Index (HPDI)), respectively. Multivariate Cox Regression models and restricted cubic spline analyses were used to assess the associations of plant‐based diets, inflammatory diets, and micronutrients with MACE and mortality among populations with CKM stages 0–3. Mediation analyses assessed the mediating effects of oxidative stress, phenotypic aging, and inflammatory markers. 82,222 and 16,842 participants were included in the analyses in UKB and NHANES, respectively. AMED and HPDI were significantly negatively associated with MACE and all‐cause mortality at higher tertiles, while DII was significantly positively associated. For each standard deviation increase in the scores of AMED and HPDI, the incidence of MACE decreased by 5%–7%. Higher intake of vitamins (Alpha/Beta‐carotene and vitamin C) and minerals (copper, iron, and magnesium) may reduce the incidence of MACE and mortality. Oxidative stress, phenotypic aging, and inflammatory markers partially mediated the associations (mediation ratio: 1.3%–29.5%). Adhering to plant‐based dietary patterns such as HPDI and AMED, reducing the intake of pro‐inflammatory red meat‐based diets, and ensuring adequate dietary intake of carotene and minerals may confer benefits for both metabolic and cardiovascular health.

## Graphical abstract

Graphical abstract illustrating the study design and core findings of two prospective cohorts (UK Biobank, *n* = 82,222; NHANES, *n* = 16,842) enrolling participants with CKM syndrome stages 0–3. We examined associations of plant‐based diets (HPDI, AMED), pro‐inflammatory diet (DII), and multiple micronutrients (vitamins, minerals) with MACE and all‐cause mortality. The underlying biological mechanisms include oxidative stress, inflammation, and phenotypic aging, which mediated 1.3%–29.5% of the associations. Plant‐rich dietary patterns reduced cardiovascular and mortality risk, while pro‐inflammatory diet elevated risk; micronutrients exerted cardioprotective effects by alleviating the above pathological pathways.

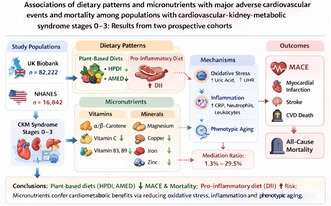

## 1 Introduction

In 2023, the American Heart Association (AHA) introduced Cardiovascular‐Kidney‐Metabolic (CKM) Syndrome, a paradigm linking cardiovascular disease (CVD), chronic kidney disease (CKD), and metabolic dysfunction into a unified, staged framework (Ndumele et al. 2023). Epidemiological data reveal that 86.4% of U.S. adults meet criteria for stage 1 or higher CKM, with 12.8% progressing to advanced stages (3–4), mirroring global trends observed in China and other aging populations (Aggarwal et al. 2024). Atherosclerosis, hypertension, coronary artery disease, and heart failure are among the cardiovascular disorders that characterize the progressive course of CVD (Casas et al. 2018). MACEs, or major adverse cardiovascular events, are brought on by these pathogenic signs. Incidents of nonfatal myocardial infarction, stroke, and fatal cardiovascular events are referred to as MACEs (Stewart et al. 2016; Li et al. 2024). This syndemic necessitates integrative interventions targeting shared pathways, particularly dietary patterns influencing metabolic health, inflammation, and aging (Stewart et al. 2016; Hariharan et al. 2022; Zhu et al. 2024; Gao et al. 2024; Chen et al. 2025). Among these, plant‐based diets (e.g., Mediterranean, healthful plant‐based diets) and pro‐inflammatory diets rich in red/processed meats emerge as critical modifiable factors, though their roles in early CKM stages (0–3) and MACEs remain incompletely understood.

Accumulating evidence underscores the cardiometabolic benefits of plant‐centric diets like the Alternative Mediterranean Diet (AMED) and Healthful Plant‐Based Diet Index (HPDI), characterized by high fruit, vegetable, legume, and whole grain intake, with meta‐analyses linking these patterns to 19%–25% reductions in CVD and CKD risk due to their anti‐inflammatory and antioxidant properties (Stewart et al. 2016; Sofi et al. 2010; Martínez‐González et al. 2019; Rees et al. 2019; Sotos‐Prieto et al. 2020; Tu et al. 2023; Shang et al. 2023). Conversely, diets high in red/processed meats, quantified by the Dietary Inflammatory Index (DII), elevate systemic inflammation and are associated with a 91% increased CVD risk in high‐DII populations, as heme iron, saturated fats, and advanced glycation end‐products (AGEs) in red meat mediate pro‐inflammatory effects that exacerbate oxidative stress and endothelial dysfunction (Liu et al. 2025). Crucially, the protective effects of plant‐based diets and the harms of pro‐inflammatory diets may operate through modulation of phenotypic aging and chronic inflammation, both hallmarks of CKM syndrome pathogenesis. Diets rich in red meat and refined carbohydrates have been shown to accelerate phenotypic aging (marked by accelerated physiological decline) and elevate biomarkers like C‐reactive protein and IL‐6, while plant‐based diets, abundant in polyphenols, fiber, and specific micronutrients (e.g., carotenoids, magnesium) that may synergize to attenuate CKM progression (though their interactions warrant further investigation), may decelerate aging by upregulating antioxidant defenses (e.g., via carotenoids) and suppressing NF‐κB‐mediated inflammation (An et al. 2022; Mutruc et al. 2025; Hu et al. 2025; Aleksandrova et al. 2021; Senoner and Dichtl 2019); however, no large‐scale studies have yet examined whether these mechanisms mediate the diet‐CKM relationship, particularly in early‐stage disease where interventions could halt progression.

To address these gaps, we leveraged data from the UK Biobank (UKB) and the US National Health and Nutrition Examination Survey (NHANES) to examine the associations of AMED, HPDI, DII, and micronutrient intake with MACE and mortality in individuals with CKM stages 0–3. We further explored the mediating roles of oxidative stress, phenotypic aging, and inflammatory markers in these associations (Liu et al. 2025). Our findings provide novel insights into the potential of dietary interventions to mitigate cardiovascular and metabolic risks in this high‐risk population.

## 2 Materials and Methods

### 2.1 Study Population and Design

This study conducted statistical analyses based on data sourced from the UKB and the NHANES. The UKB is a large‐scale biomedical research project covering the entire United Kingdom, which collected genetic data, lifestyle information, biological samples, and health records from 500,000 participants. For this study, data from 2006 to 2010 were selected. After excluding individuals with incomplete dietary information, missing outcome indicators, incomplete covariates, or those who had progressed to stage 4 of CKM syndrome, 82,222 subjects were ultimately included (Figure S1). The NHANES database is a nationally representative health and nutrition survey in the United States. This study incorporated data from 2005 to 2018, and after applying exclusion criteria, 16,842 participants met the analytical standards (Figure S2). All participants provided written informed consent.

### 2.2 Definition of Plant‐Based Diets, Inflammatory Diets, and Micronutrients

The NHANES and UKB adopted distinct dietary assessment methods for data collection and analysis. In the NHANES project, almost all participants completed two 24‐h dietary recall assessments: an initial face‐to‐face interview at the Mobile Examination Center (MEC) and a follow‐up telephone interview conducted 3–10 days later. This study used the average values of the two surveys as the final dietary intake assessment. For UKB, detailed 24‐h dietary information was obtained through electronic questionnaires and the web‐based automated 24‐h dietary recall system (Oxford WebQ).

Regarding micronutrient analysis, this study focused on 8 minerals and 12 vitamins, which play significant roles in cardiometabolic health. Furthermore, the study incorporated three dietary patterns. Based on the different dietary assessment methods and data characteristics of each database, two dietary patterns in the NHANES were selected, including DII and AMED. While HPDI and AMED were used for comprehensive assessment in the UKB. Specific details of different dietary scores are as follows: Table S1.

### 2.3 Alternative Mediterranean Diet (AMED) Score

In this study, the AMED score was utilized to evaluate participants' adherence to the Mediterranean dietary pattern. The score quantifies dietary intake across nine pivotal components, employing sex‐specific median values as the dividing thresholds. For components that are considered beneficial to health (e.g., vegetables, fruits, whole grains), participants were assigned 1 point if their intake exceeded the sex‐specific median, while intake below the median received 0 points. Conversely, for potentially harmful components (such as the ratio of monounsaturated to saturated fatty acids, red/processed meats, and poultry), reverse scoring was applied (below median = 1 point; above = 0). Total scores ranged from 0 to 9, with higher scores indicating stronger alignment with Mediterranean diet principles (Yin et al. 2021).

### 2.4 Healthful Plant‐Based Diet Index (HPDI)

The HPDI assessed dietary quality using 17 food groups that were categorized into three distinct types, including healthful plant‐based foods (e.g., whole grains, fruits/vegetables), unhealthful plant‐based foods (e.g., refined grains, sugary foods), and animal‐derived foods (meat, fish, dairy, eggs). This pattern assigned positive weights (1–5 points by fifth percentiles) to healthful plant foods, while unhealthful plant and animal foods received inverse weights. Total scores theoretically ranged from 17 to 85, with higher values reflecting greater adherence to a healthful plant‐based diet (Schorr et al. 2025). Notably, vegetable oil was excluded from UKB calculations due to data limitations.

### 2.5 Dietary Inflammatory Index (DII)

The DII quantified diet‐associated inflammation by evaluating 45 food components with pro‐/anti‐inflammatory properties, validated against six inflammatory biomarkers (IL‐1β, IL‐4, IL‐6, IL‐10, TNF‐α, CRP). The score > 0 indicates pro‐inflammatory effects, whereas score < 0 suggests anti‐inflammatory effects, with magnitude reflecting effect strength. For NHANES, 28 of 45 components were analyzed due to questionnaire constraints (Hariharan et al. 2022).

### 2.6 Definition of CKM Syndrome Stage

CKM Stage 0 included participants with normal body mass index (BMI) (< 23 kg/m^2^ for individuals with Asian ethnicity and < 25 kg/m^2^ for other racial and ethnic groups), normal waist circumference (< 80 and < 90 cm for women and men with Asian race, respectively, and < 88 and < 102 cm for women and men in all other race and ethnicity categories, respectively) who did not meet criteria for the other stages. CKM Stage 1 identified individuals with an elevated BMI (≥ 23 kg/m^2^ for individuals with Asian race and > 25 kg/m^2^ for all other race and ethnic groups), an elevated waist circumference (≥ 80 and ≥ 90 cm for women and men with Asian race, respectively, and ≥ 88 and ≥ 102 cm for women and men in other race and ethnicity categories, respectively), or prediabetes (defined as a glycated hemoglobin of 5.7% to < 6.5% or a fasting blood glucose of 100 mg/dL to < 126 mg/dL). CKM Stage 2 identified participants with metabolic risk factors or moderate‐to‐high‐risk CKD per Kidney Disease Improving Global Outcomes (KDIGO) criteria, as recommended by the AHA. Qualifying metabolic risk factors included elevated fasting serum triglycerides (≥ 135 mg/dL), hypertension, diabetes, or metabolic syndrome (≥ 3 of the following: elevated waist circumference, low high density lipoprotein cholesterol (HDL) level [< 40 mg/dL or < 50 mg/dL for men or women, respectively], fasting serum triglycerides ≥ 150 mg/dL, elevated blood pressure [systolic blood pressure ≥ 130, diastolic blood pressure ≥ 80 mmHg, and/or use of blood pressure‐lowering medications], or prediabetes). CKD stages were identified based on GFR and urinary albumin‐to‐creatinine ratio. CKM Stage 3 was identified based on the presence of very‐high‐risk KDIGO CKD stages or a high‐predicted 10‐year CVD risk. 10‐year cardiovascular risk was estimated with the AHA Predicting Risk of CVD EVENTs (PREVENT) equations (Stewart et al. 2016). High risk was defined as ≥ 20% 10‐year CVD risk (based on recommended thresholds). The PREVENT equations were developed and validated for adults 30–79 years of age. As such, risk was not estimated for adults < 30 years. However, to minimize underestimation of CKD Stage 3, adults ≥ 80 years were not excluded from 10‐year CVD risk. Instead, adults ≥ 80 years were assigned an age of 79 years when determining 10‐year CVD risk to allow for conservative estimates. Further, PREVENT was developed for variables with the following ranges: total cholesterol 130–320 mg/dL, HDL 20–100 mg/dL, systolic blood pressure 90–200 mmHg, and GFR 14–140 mL/min/1.73 m^2^. To approximate PREVENT risk strata, values for these variables above or below these bounds were set to the upper or lower bounds of allowable values respectively (e.g., total cholesterol of 330 mg/dL was set as 320 mg/dL). Cardiac biomarkers and cardiovascular imaging were not available to identify subclinical CVD. CKM Stage 4 was identified based on self‐reported established cardiovascular disease (coronary heart disease, angina, heart attack, heart failure, and stroke). Atrial fibrillation and peripheral artery disease were not included, as these data were not available (Ndumele et al. 2023).

### 2.7 Definition of Major Adverse Cardiovascular Events (MACE) and All‐Cause Mortality

For the UKB cohort, data on outcomes were obtained through electronic linkage with hospital admission records in England, Wales, and Scotland. Mortality data in NHANES were derived from the National Death Index (NDI) linkage files. Death from any cause was referred to as all‐cause mortality, but cause‐specific mortality emerged utilizing the International Classification of Diseases, Tenth Revision (ICD‐10) codes, with CVD mortality specifically identified by ICD‐10 codes I00‐I09, I11, I13, I20‐I51, and I60‐I69. MACE was defined as a composite endpoint consisting of CVD mortality, non‐fatal myocardial infarction (MI), and stroke (Xie et al. 2025).

### 2.8 Definition of Oxidative Stress, Phenotypic Aging, and Inflammatory Markers

Both NHANES and UKB used standardized biomarker measurement protocols for laboratory analyses. C‐reactive protein (CRP) was quantitatively determined in blood samples using immunoturbidimetric assays. Hematologic parameters, including neutrophil, leukocyte, and lymphocyte counts, were measured using automated hematology analyzers. Oxidative stress biomarkers included uric acid (UA) and UA/HDL‐C ratio (UHR). All these biomarkers were treated as continuous variables in our mediation analyses (Liu et al. 2025).

Phenotypic age was calculated using a composite algorithm incorporating 10 aging‐related biomarkers $\left(\text{phenotypic}\;\mathrm{age}=141.50225+\frac{\text{In}\left[–0.00553\times \text{In}\left(1–\text{mortality risk}\right)\right]}{0.09016}\right)$. Phenotypic age acceleration was assessed by calculating the residuals of phenotypic age regressed on chronological age using linear regression. Individuals exhibiting positive residuals (phenotypic age > chronological age) were defined as having accelerated phenotypic aging, whereas those with negative residuals (phenotypic age < chronological age) were defined as having decelerated phenotypic aging (Yang et al. 2022).

### 2.9 Covariates

The study adjusted for the following covariates in the two cohorts: age (continuous), sex (male/female), race (UKB: White, Asian/Asian British, Black/Black British, Chinese, Mixed, Other; NHANES: Mexican American, Other Hispanic, Non‐Hispanic White, Non‐Hispanic Black, Other Race including Multiracial), educational attainment (less than high school/high school or above), BMI (continuous), total energy intake (continuous), socioeconomic status indicators (UKB: Townsend deprivation index in tertiles T1–T3; NHANES: family income‐to‐poverty ratio categorized as < 1.3, 1.3–3.5, or ≥ 3.5), lifestyle factors (smoking status: yes/no; alcohol consumption: continuous; physical activity level: adequate/inadequate), and medical history (history of diabetes: yes/no).

### 2.10 Statistical Analysis

In the baseline characteristics, continuous variables were presented as mean ± standard deviation (SD), while categorical variables were described using counts (percentages). Group comparisons were performed using *t*‐tests for continuous variables and *χ* ^2^ tests for categorical variables. The study employed multidimensional statistical models to explore the correlations between dietary factors and corresponding results. For NHANES analyses, the complex multistage probability sampling design was fully accounted for in all statistical models. A 14‐year combined sample weight was constructed across the seven consecutive survey cycles (2005–2018) following the standard analytical guidelines recommended by the National Center for Health Statistics, whereby the 2‐year cycle weight was divided by the total number of cycles (7) included in the analysis. All Cox regression models incorporated the appropriate sample weights, strata, and primary sampling units to produce nationally representative estimates with correctly estimated standard errors. Firstly, Cox proportional hazards models were constructed with the first tertile of each dietary indicator as the reference. Two adjusted models were implemented: Model 1 adjusted for age, sex, race, income level, education level, smoking status, alcohol consumption, physical activity, and total energy intake; Model 2 additionally adjusted for BMI and diabetes history. Results were reported as hazard ratios (HRs) with 95% confidence intervals (CIs). The proportional hazards assumption was formally verified for all Cox regression models using Schoenfeld residuals, and no violations were identified for any covariate included in the models. Secondly, restricted cubic spline (RCS) analyses with knots at the 5th, 50th, and 95th percentiles were used to evaluate nonlinear relationships. A statistically significant nonlinear association was determined when the *p* value for nonlinearity was < 0.05.

Furthermore, the purpose of the mediation analysis was to investigate the mediating impacts of oxidative stress, phenotypic aging, and inflammatory markers. Mediation analyses were performed using the CAUSALMED procedure in SAS (version 9.4), which implements a counterfactual‐based causal mediation framework. This approach decomposes the total effect into natural direct and indirect effects, accommodates potential exposure‐mediator interactions, and provides robust estimates of the proportion mediated. All mediation models were adjusted for the same covariate set as the primary Cox regression models, with the complex survey design accounted for throughout. Finally, stratified analyses by sex were performed to assess the interaction of sex on exposure and outcomes. All statistical analyses were performed using R software (version 4.2.2) and SAS software (version 9.4), with a two‐sided significance level of *p* < 0.05.

## 3 Results

### 3.1 Baseline Characteristics of Participants From UKB and NHANES

Our analysis included 82,222 participants from UKB and 16,842 from NHANES with CKM stages 0–3 (Table 1). Compared to NHANES participants, UKB participants were older (mean 55.9 vs. 47.9 years), had fewer males (45.4% vs. 47.5%), a higher proportion of White individuals (97.0% vs. 55.1%), lower BMI (26.5 vs. 29.1 kg/m^2^), and higher total energy intake (2084.0 vs. 2061.7 kcal/day). NHANES participants had higher educational attainment and higher AMED scores. Mean HPDI and DII scores were 56.8 (SD: 6.5) and 1.1 (SD: 1.7), respectively. Significant between‐cohort differences were observed in micronutrient intake.

**TABLE 1:** Baseline characteristics of participants in the UK Biobank and US NHANES.

Characteristics	UK Biobank (*N* = 82,222)	US NHANES (*N* = 16,842)	*p*
*Demographics*			
Age, mean (SD), years	55.9 (7.8)	47.9 (16.9)	< 0.001
Male, *n* (%)	37,313 (45.4)	7992 (47.5)	< 0.001
White, *n* (%)	79,716 (97.0)	9272 (55.1)	< 0.001
Less than high school, *n* (%)	18,993 (23.1)	3652 (21.7)	< 0.001
Low household income^a^, *n* (%)	9452 (11.5)	5145 (30.6)	< 0.001
Inadequate physical activity, *n* (%)	2672 (3.3)	10,903 (64.7)	< 0.001
Non‐smoking, *n* (%)	46,921 (57.1)	7203 (42.8)	< 0.001
Alcohol consumption, mean (SD), g/day	17.5 (17.3)	8.3 (20.4)	< 0.001
History of diabetes, *n* (%)	3108 (3.8)	1750 (10.4)	< 0.001
BMI, mean (SD), kg/m^2^	26.5 (4.4)	29.1 (6.9)	< 0.001
Total energy intake, mean (SD), kcal	2084.0 (655.9)	2061.7 (821.9)	< 0.001
*Dietary patterns scores (SD)*			
AMED	4.2 (1.3)	5.7 (1.0)	< 0.001
DII	—	1.1 (1.7)	—
HPDI	56.8 (6.5)	—	—
*Vitamins (SD)*			
Retinol (Vitamin A), mcg	472.3 (1107.7)	409.3 (387.5)	< 0.001
Alpha‐carotene, mcg	524.9 (770.4)	425.4 (1050.4)	< 0.001
Beta‐carotene, mcg	2686.3 (2868.5)	2267.5 (3702.0)	< 0.001
Thiamin (Vitamin B1), mg	1.9 (0.8)	1.6 (0.8)	< 0.001
Riboflavin (Vitamin B2), mg	1.9 (0.7)	2.1 (1.1)	< 0.001
Niacin (Vitamin B3), mg	38.4 (13.3)	25.3 (12.7)	< 0.001
Vitamin B6, mg	2.1 (0.8)	2.1 (1.3)	< 0.001
Folate (Vitamin B9), mcg	316.9 (119.6)	402.1 (215.8)	< 0.001
Vitamin B12, mcg	6.2 (3.9)	5.0 (4.7)	< 0.001
Vitamin C, mg	131.6 (87.2)	85.2 (78.8)	< 0.001
Vitamin D, mcg	3.6 (3.5)	4.7 (4.5)	< 0.001
Vitamin E, mg	11.1 (5.3)	8.1 (5.5)	< 0.001
*Minerals (SD)*			
Calcium, mg	991.8 (386.9)	934.2 (497.9)	< 0.001
Phosphorus, mg	1437.2 (432.5)	1349.2 (572.0)	< 0.001
Magnesium, mg	338.8 (105.2)	296.4 (130.5)	< 0.001
Iron, mg	12.6 (4.3)	14.8 (7.6)	< 0.001
Zinc, mg	9.8 (3.8)	11.2 (6.3)	< 0.001
Copper, mg	1.4 (0.6)	1.3 (0.8)	< 0.001
Sodium, mg	1958.0 (904.9)	3431.6 (1498.9)	< 0.001
Selenium, mcg	52.8 (28.7)	113.3 (53.2)	< 0.001

*Note:* Descriptive data were shown as mean (SD) while categorical variables were reported as *n* (%).

Abbreviations: AMED, Alternate Mediterranean Diet; BMI, body mass index; DII, Dietary Inflammation Index; HPDI, Healthful Plant‐Based Diet Index; *N*, number; NHANES, National Health and Nutrition Examination Survey; SD, standard deviation.

^a^ In NHANES, low household income referred to family poverty/income ratio of ≤ 1.3; In UK Biobank, household income < £18,000 was defined as low household income. *p* value < 0.05 were considered significant. —: data not available.

### 3.2 Associations of Plant‐Based Diets, Inflammatory Diets With MACE and Mortality

**FIGURE 1:**
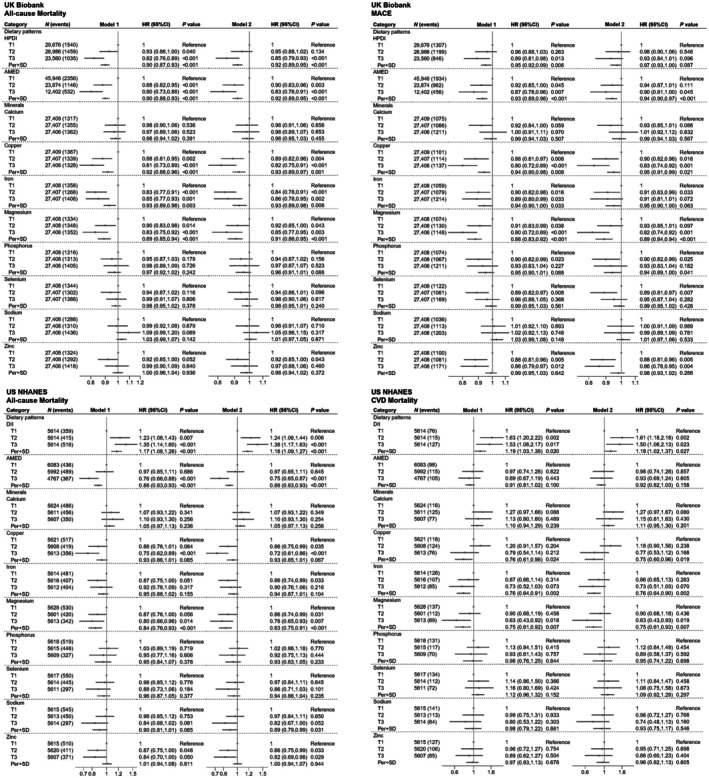
Association of plant‐based diets, inflammatory diets and minerals with major adverse cardiovascular events and mortality. Model 1: age (continuous), sex (male or female), ethnicity/race (UKB: White, Asian or Asian British, Black or Black British, Chinese, Mixed, Other ethnic group; NHANES: Mexican American, Other Hispanic, Non‐Hispanic White, Non‐Hispanic Black, Other Race—Including Multi‐Racial), total energy intake (continuous), educational level (less than high school, high school and above), Townsend deprivation index (T1, T2, T3) in UKB, ratio of family income to poverty (< 1.3, 1.3–3.5, ≥ 3.5) in NHANES, smoking status (Yes or No), alcohol consumption (continuous), physical activity (adequate, inadequate). Model 2: Model 1 + BMI (continuous), history of diabetes (Yes or No). *p*‐values less than 0.05 (*p* < 0.05) were considered significant. AMED, Alternate Mediterranean Diet; BMI, body mass index; CI, confidence interval; CVD, cardiovascular disease; DII, Dietary Inflammation Index; HPDI, Healthful Plant‐Based Diet Index; HR, hazard ratio; MACE, major adverse cardiovascular events; *N*, number; NHANES, National Health and Nutrition Examination Survey; SD, standard deviation; UKB, UK Biobank.

As illustrated in Figure 1, AMED, HPDI, and DII exhibited significant associations with MACE and all‐cause mortality. In UKB, a one‐SD increase in each of HPDI and AMED scores was associated with a 5%–10% reduction in the incidence of MACE and all‐cause mortality (MACE: HPDI: HR: 0.95, 95% CI: (0.92, 0.99); AMED: 0.93 (0.89, 0.96); all‐cause mortality: HPDI: 0.90 (0.87, 0.93); AMED: 0.90 (0.88, 0.93)). In US NHANES, a one‐SD increase in AMED scores was associated with a 12% reduction in the incidence of all‐cause mortality. However, a one‐SD increase in DII was also associated with an 18% increased risk of both all‐cause mortality (1.18 (1.09, 1.27)) and CVD mortality (1.18 (1.02, 1.37)). Furthermore, compared to the lowest tertile, the highest tertile of HPDI and AMED scores significantly reduced the incidence of MACE by 11% and 13% in UKB, respectively. Participants in the highest tertile of DII scores had 38% higher all‐cause mortality (1.38 (1.17, 1.63)) and 50% higher CVD mortality (1.50 (1.06, 2.13)) compared to the lowest tertile.

In addition to this, as shown in Figures S3–, S5, we further evaluated the association of HPDI and AMED with CVD mortality, stroke, and MI in UKB. Per SD increase in HPDI score, the risk of CVD mortality decreased by 10% (0.90 (0.83, 0.98)), while each SD increase in AMED score was associated with an 8% reduction in CVD mortality risk (0.92 (0.85, 1.00)). Separately, each SD increase in HPDI conferred a 6% lower stroke risk (0.94 (0.89, 0.99)), and each SD increase in AMED corresponded to a 9% reduction in MI risk (0.91 (0.86, 0.95)).

### 3.3 Subgroup Analysis of Sex

To evaluate sex‐specific effects, we conducted subgroup analyses stratified by sex in both cohorts (Tables S2–, S8). In UKB participants with CKM stage 0–3, AMED scores demonstrated a significant multiplicative interaction with sex on MACE risk (*p* value = 0.038). Across all adjusted models, higher AMED scores were associated with lower MACE risk in males (HR range: 0.78–0.90), but this protective association was non‐significant in females. No other significant multiplicative interactions were observed between sex and dietary pattern scores (AMED, DII, or HPDI) for any outcome. Notably, the directionality of protective associations for HPDI and AMED remained consistent with the overall cohort in sex‐stratified analyses.

### 3.4 Association of Minerals Intake With MACE and Mortality

Figures 1 and S3–, S5 illustrate the associations of mineral intake with outcome. In both cohorts, higher intakes of copper, iron, and magnesium were significantly associated with reduced risks of MACE, all‐cause and CVD mortality (T3 vs. T1: HR range: 0.63–0.89). Selenium, zinc, and phosphorus intake also demonstrated protective associations against MACE, though effect magnitudes varied. Specifically, elevated magnesium and copper intake significantly lowered MI risk (T3 vs. T1: Mg: 0.73 (0.62, 0.86); Cu: 0.80 (0.68, 0.94)), while only higher zinc intake was associated with reduced stroke risk (T2 vs. T1: 0.84 (0.74, 0.96); T3 vs. T1: 0.85 (0.73, 0.99)).

### 3.5 Association of Vitamin Intake With MACE and Mortality

**FIGURE 2:**
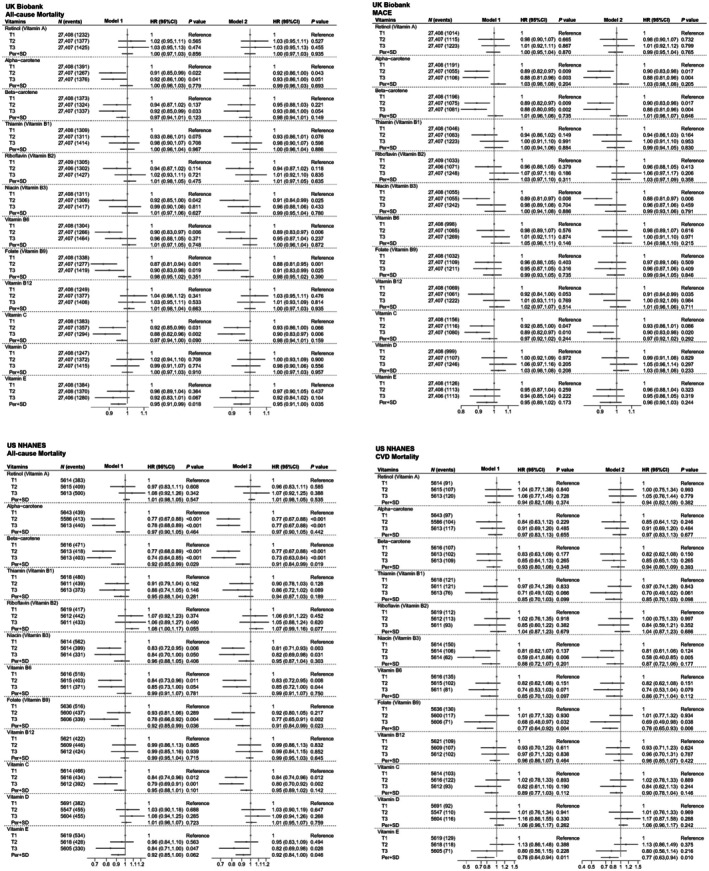
Association of vitamins with major adverse cardiovascular events and mortality. Model 1: age (continuous), sex (male or female), ethnicity/race (UKB: White, Asian or Asian British, Black or Black British, Chinese, Mixed, Other ethnic group; NHANES: Mexican American, Other Hispanic, Non‐Hispanic White, Non‐Hispanic Black, Other Race—Including Multi‐Racial), total energy intake (continuous), educational level (less than high school, high school and above), Townsend deprivation index (T1, T2, T3) in UKB, ratio of family income to poverty (< 1.3, 1.3–3.5, ≥ 3.5) in NHANES, smoking status (Yes or No), alcohol consumption (continuous), physical activity (adequate, inadequate). Model 2: Model 1 + *BMI* (continuous), history of diabetes (Yes or No). *p*‐values less than 0.05 (*p* < 0.05) were considered significant. BMI, body mass index; CI, confidence interval; CVD, cardiovascular disease; HR, hazard ratio; MACE, major adverse cardiovascular events; *N*, number; NHANES, National Health and Nutrition Examination Survey; SD, standard deviation; UKB, UK Biobank.

Figures 2 and S6–, S8 illustrate the associations of vitamin intake with outcome. In both cohorts, higher intakes of alpha‐carotene, beta‐carotene, and VC were significantly associated with reduced risks of MACE, all‐cause and CVD mortality (T3 vs. T1: HR range: 0.73–0.92). In UKB, compared to the lowest tertile, the highest tertile of alpha‐carotene was associated with a 12% lower stroke risk (0.88 (0.77, 0.99)), beta‐carotene with a 12% reduced MI risk (0.88 (0.78, 0.99)), and VC with a 20% decreased CVD mortality risk (0.80 (0.66, 0.97)). In NHANES, higher levels of VB3 and VB9 intake were associated with a lower risk of all‐cause and CVD mortality (T3 vs. T1: VB3: 0.82 (0.69, 0.98)/0.58 (0.40, 0.85); VB9: 0.77 (0.65, 0.91)/0.69 (0.49, 0.98)). For each SD increase in VE, the risk of all‐cause and CVD mortality decreased by 9% and 23%, respectively (all‐cause: 0.92 (0.84, 1.00); CVD: 0.77 (0.63, 0.94)).

### 3.6 Associations of Continuous HPDI, AMED, and DII With MACE and Mortality

**FIGURE 3:**
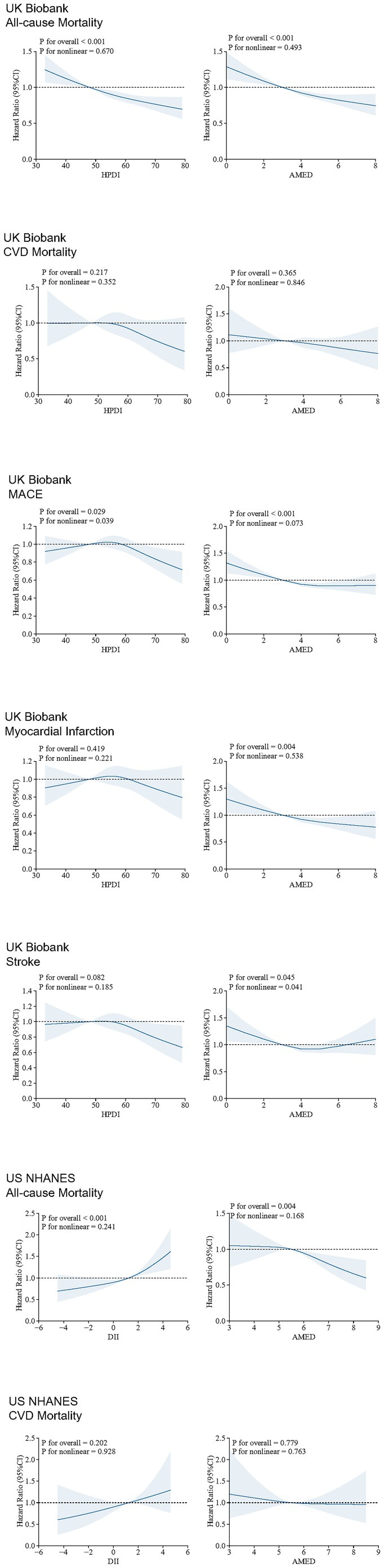
Restricted cubic spline plots of the association of dietary patterns scores with major adverse cardiovascular events and mortality. Model: age (continuous), sex (male or female), ethnicity/race (UKB: White, Asian or Asian British, Black or Black British, Chinese, Mixed, Other ethnic group; NHANES: Mexican American, Other Hispanic, Non‐Hispanic White, Non‐Hispanic Black, Other Race—Including Multi‐Racial), total energy intake (continuous), educational level (less than high school, high school and above), Townsend deprivation index (T1, T2, T3) in UKB, ratio of family income to poverty (< 1.3, 1.3–3.5, ≥ 3.5) in NHANES, smoking status (Yes or No), alcohol consumption (continuous), physical activity (adequate, inadequate), BMI (continuous), history of diabetes (Yes or No). *p*‐values less than 0.05 (*p* < 0.05) were considered significant. AMED, Alternate Mediterranean Diet; BMI, body mass index; CI, confidence interval; DII, Dietary Inflammation Index; HPDI, Healthful Plant‐Based Diet Index; HR, hazard ratio; MACE, major adverse cardiovascular events; NHANES, National Health and Nutrition Examination Survey; UKB, UK Biobank.

Figure 3 depicts the continuous associations of dietary pattern scores with MACE and mortality in both cohorts. In both cohorts, no significant nonlinear associations were observed between any dietary pattern score (HPDI, AMED, DII) and all‐cause, CVD mortality, or MI. Notably, HPDI exhibited a significant inverted U‐shaped nonlinear association with MACE risk (*P* for nonlinear = 0.039). In contrast, AMED demonstrated a U‐shaped nonlinear association with stroke risk (*P* for nonlinear = 0.041).

### 3.7 The Mediating Role of Oxidative Stress, Phenotypic Aging, and Inflammatory Markers

**FIGURE 4:**
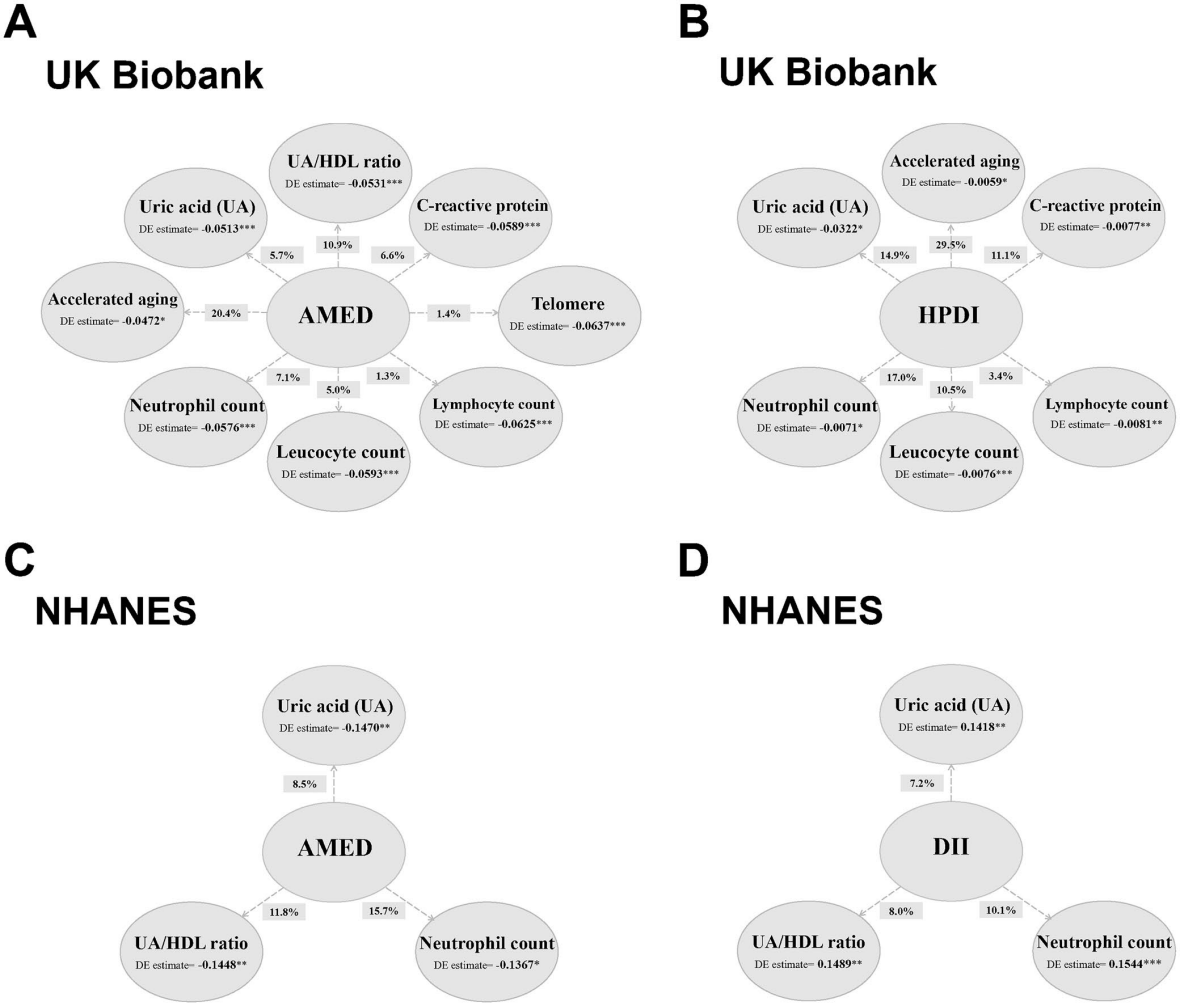
Oxidative stress, phenotypic aging, and inflammatory markers mediate the association of AMED, HPDI, and DII with major adverse cardiovascular events. (A) UKB: AMED to MACE; (B) UKB: HPDI to MACE; (C) NHANES: AMED to CVD Mortality; (D) NHANES: DII to CVD Mortality. Model: age (continuous), sex (male or female), ethnicity/race (UKB: White, Asian or Asian British, Black or Black British, Chinese, Mixed, Other ethnic group; NHANES: Mexican American, Other Hispanic, Non‐Hispanic White, Non‐Hispanic Black, Other Race—Including Multi‐racial), total energy intake (continuous), educational level (less than high school, high school and above), Townsend deprivation index (T1, T2, T3) in UKB, ratio of family income to poverty (< 1.3, 1.3–3.5, ≥ 3.5) in NHANES, smoking status (Yes or No), alcohol consumption (continuous), physical activity (adequate, inadequate), BMI (continuous), history of diabetes (Yes or No). In the UKB cohort, MACE (comprising CVD mortality, non‐fatal myocardial infarction, and stroke) was used as the mediation outcome. In the NHANES cohort, CVD mortality was used as the outcome, as non‐fatal cardiovascular events could not be reliably ascertained through the available follow‐up data infrastructure. Only statistically significant mediation pathways are presented. *p value < 0.05, **p value < 0.01, ***p value < 0.001. AMED, Alternate Mediterranean Diet; BMI, body mass index; CVD, cardiovascular disease; DE, direct effect; DII, Dietary Inflammation Index; HPDI, Healthful Plant‐Based Diet Index; MACE, major adverse cardiovascular events; NHANES, National Health and Nutrition Examination Survey; UKB, UK Biobank.

In this study, mediation analyses were conducted across the two cohorts to explore the potential mediating roles of oxidative stress, phenotypic aging, and inflammatory markers. As shown in Figure 4, in the UKB cohort, these mediating variables partially mediated the associations between HPDI/AMED and MACE, with mediation ratios as follows: phenotypic aging (29.5%/20.4%), telomere (1.4%), UA (14.9%/5.7%), UHR (10.9%), leukocytes (10.5%/5.0%), neutrophils (17.0%/7.1%), lymphocyte (3.4%/1.3%), and CRP (11.1%/6.6%). In the NHANES cohort, given that non‐fatal cardiovascular events could not be reliably ascertained, CVD mortality was used as the outcome for mediation analyses. UA, UHR, and neutrophils partially mediated the associations between DII/AMED and CVD mortality, with mediation ratios of UA (7.2%/8.5%), UHR (8.0%/11.8%), and neutrophils (10.1%/15.7%).

## 4 Discussion

This observational study, leveraging two large prospective cohorts, is among the first to evaluate associations of plant‐based diets, pro‐inflammatory diets, and micronutrients with MACE and all‐cause mortality in individuals with CKM syndrome stages 0–3. We demonstrated that greater adherence to healthful plant‐based dietary patterns (AMED and HPDI) was significantly associated with lower risks of MACE and all‐cause mortality. Conversely, pro‐inflammatory diets reflected by higher DII correlated with increased risks of all‐cause and CVD mortality. Notably, there was a significant nonlinear association between HPDI and MACE risk. Oxidative stress, phenotypic aging, and inflammatory markers partially mediated these associations.

Consistent with prior research, greater adherence to HPDI and AMED scores was associated with enhanced cardiovascular benefits (Li et al. 2024; Sofi et al. 2010; Akesson et al. 2014). While the HPDI exhibited a significant nonlinear relationship with MACE risk, exceeding a score of 60 conferred progressively lower MACE risk. This inverse association between high‐quality diets (AMED/HPDI) and reduced CVD incidence aligns with findings from several large cohorts. Notably, a study reported HRs for CVD that were 17% and 14% lower in the highest versus lowest quintiles of AMED and HPDI scores, respectively. Further supporting this, a long‐term cohort study (follow‐up up to 32 years) linked high diet quality (AMED/HPDI) with an 8%–21% reduction in risk of incidence CVD. Within our specific population (CKM stages 0–3), participants in the highest tertile of HPDI and AMED scores demonstrated 11% and 13% lower MACE risks, respectively, compared to those in the lowest tertile. Additionally, both cohorts revealed significant reductions (17%–20%) in all‐cause mortality risk associated with higher AMED and HPDI adherence. The observed sex differences in the protective effect of AMED against MACE may be driven by several factors. Biologically, premenopausal women benefit from the cardioprotective effects of endogenous estrogen, which might mask the incremental cardiovascular benefits of dietary interventions during earlier life stages. Behaviorally, women in our cohorts generally exhibited higher baseline adherence to healthy dietary patterns and lower rates of smoking and alcohol consumption compared to men, potentially creating a ‘ceiling effect’ where further dietary improvements yield smaller marginal benefits. Although MACE analyses were restricted to the UKB cohort due to the unavailability of non‐fatal cardiovascular event data in NHANES, cross‐cohort comparability is maintained for mortality endpoints. CVD mortality, which is a constituent component of the MACE composite, was available in both cohorts and demonstrated consistent directional associations with dietary exposures across UKB and NHANES, providing complementary support for the cardiovascular mortality component of the primary composite findings. All‐cause mortality associations were likewise consistent across cohorts, further supporting the robustness of the overall findings.

The cardioprotective effects of these dietary patterns likely stem from their shared emphasis on plant‐based foods, healthy fats, and limited intake of refined carbohydrates and processed meats—components collectively linked to improved metabolic and inflammatory profiles (Shan et al. 2020; Movassagh and Vatanparast 2017; Bach et al. 2019). This is reinforced by an international cohort study of coronary heart disease patients, which found that higher Mediterranean diet adherence correlated with lower MACE risk (Stewart et al. 2016). Higher intake of whole grains, fish, and foods rich in monounsaturated fatty acids appears to confer cardiovascular benefits across diverse populations, including both healthy individuals and those with obesity.

Conversely, pro‐inflammatory diets may exacerbate chronic low‐grade inflammation (Huang et al. 2024). Our findings corroborate this, showing a positive association between the DII and risks of both all‐cause and CVD mortality. Specifically, each standard deviation increase in DII score elevated these risks by 18%. The DII comprehensively captures the inflammatory potential of an individual's diet, and its positive association with CVD mortality highlights the prognostic significance of dietary inflammation. Collectively, these results highlight the significant public health impact of adhering to healthy dietary patterns, which can reduce the substantial financial strain of CVD and markedly enhance one's quality of life (Bach et al. 2019; Hu et al. 2021).

Regarding the association regarding individual nutrient intake and MACE, our results offer insights into potential mechanisms underlying the observed dietary pattern effects. Higher intakes of specific vitamins (α/β‐carotene, vitamins B3, B12, and C) and minerals (selenium, phosphorus, magnesium, iron, zinc, and copper) were associated with lower MACE risk. These nutrients exert beneficial effects on cardiovascular function through diverse pathways. Observational and experimental evidence suggests antioxidants (e.g., β‐carotene, vitamin C, selenium) can mitigate inflammation, oxidative stress, and endothelial dysfunction (Hu et al. 2025). Previous studies also link higher vitamin C and zinc intake to reduced mortality risk in CVD patients (Aleksandrova et al. 2021). Vitamin C acts as a potent antioxidant, protecting against oxidative damage and LDL cholesterol oxidation, thereby maintaining vascular health (Lykkesfeldt et al. 2000). Zinc is essential for endothelial integrity, functioning as an antioxidant and membrane stabilizer while modulating nitric oxide (NO) signaling pathways to support vascular homeostasis (Zalewski et al. 2019). It is further important to note that dietary exposures examined in this study are inherently interrelated. Individuals adhering to plant‐based dietary patterns tend to simultaneously consume higher levels of protective micronutrients and maintain lower dietary inflammatory potential, creating a degree of interdependence among the dietary variables. Although dietary pattern scores and individual micronutrient analyses were conducted in separate models to minimize direct collinearity, the observed associations for specific micronutrients may partly reflect the broader dietary pattern context in which they are embedded rather than independent nutrient‐specific effects.

High adherence to anti‐inflammatory dietary patterns like AMED or HPDI reduces systemic inflammation and improves metabolic disorders (e.g., obesity, diabetes), consequently lowering severe MACE incidence (Sotos‐Prieto et al. 2020). These dietary patterns promote the intake of essential micronutrients and bioactive compounds, thereby improving cardiometabolic health, attenuating cellular oxidative stress, and potentially decelerating biological aging processes (Shang et al. 2023). These findings are further contextualized by recent CKM‐specific evidence demonstrating that elevated systemic inflammatory response index (SIRI) and advanced CKM stages are independently and jointly associated with increased all‐cause and CVD mortality in a large NHANES‐based prospective cohort, with the highest risk observed among individuals with both conditions (Cao et al. 2025). This evidence reinforces the biological plausibility of systemic inflammation as a key prognostic pathway in CKM syndrome and supports the mediating role of inflammatory markers identified in the present study. Our mediation analyses indicate that oxidative stress markers (UA and UHR; mediation ratio 5.7%–14.9%), inflammatory markers (3.4%–17.0%), and accelerated aging (1.4%–29.5%) partially mediate the relationship between dietary pattern scores and MACE risk. This indicates that the positive impacts of these dietary patterns arise partly through attenuation of inflammation and deceleration of aging. The estimated mediation proportions should therefore be interpreted as exploratory mechanistic evidence providing biological plausibility rather than definitive causal estimates. Randomized dietary intervention studies incorporating these biomarkers as intermediate endpoints would be required to establish causal mediation pathways with greater certainty.

Furthermore, it is crucial to interpret the findings regarding individual micronutrients from a food‐based perspective. In reality, micronutrients operate synergistically and antagonistically within a complex ‘food matrix’, making their tandem effects highly intricate to fully isolate. Our analyses of individual vitamins and minerals were intended as exploratory observations to identify potential nutritional drivers underlying the benefits of plant‐based diets, rather than to establish causal nutrient‐to‐nutrient interactions. Therefore, the observed protective associations of specific micronutrients should be viewed as markers of a high‐quality, nutrient‐dense diet rather than an endorsement of isolated artificial supplementation. These exploratory findings ultimately reinforce the clinical relevance of adhering to comprehensive dietary patterns, such as AMED and HPDI, which inherently account for the synergistic benefits of multiple whole foods and their constituent nutrients. AMED provides olive oil polyphenols and dietary fiber, while HPDI offers antioxidants from fruits/vegetables and magnesium from leafy greens. Polyphenols and antioxidants help maintain a youthful cellular state by preserving telomere length and modulating epigenetic clocks. Dietary fiber and omega‐3 fatty acids reshape inflammatory responses by inhibiting deleterious pathways (e.g., NLRP3 inflammasome activation) and facilitating resolution (Yan et al. 2013). Collectively, these components enhance metabolic health by improving insulin sensitivity, boosting mitochondrial function, and protecting the vasculature. Notably, higher dietary pattern scores were associated with more favorable biomarkers of aging, oxidative stress, and inflammation, suggesting that holistic dietary models may simultaneously modulate multiple molecular pathways relevant to CKM health, including both biological aging and chronic inflammation (Casas et al. 2018; Hariharan et al. 2022; Gao et al. 2024; Aleksandrova et al. 2021; Senoner and Dichtl 2019; Malesza et al. 2021). These findings are consistent with the potential value of integrated dietary strategies targeting these synergistic mechanisms for cardiovascular risk reduction, though confirmation from intervention studies is needed.

Our study represents a significant advancement in understanding the relationship between dietary patterns and the risk of MACE and individual mortality rate with CKM syndrome stages 0–3. This comprehensive investigation provides three key contributions to the field: First, it offers the most thorough evaluation to date of various established plant‐based diets, pro‐inflammatory dietary patterns, and micronutrients in relation to MACE, thereby addressing critical knowledge gaps in nutritional epidemiology. Second, by leveraging data from two large prospective cohorts (NHANES and UKB) comprising nearly 100,000 participants, our analysis provides substantial statistical power to detect associations of potential clinical relevance. Third, beyond examining simple correlations, the study provides mechanistic insights by investigating important biological mediators such as phenotypic aging markers, oxidative stress parameters, and inflammatory biomarkers—findings that hold significant implications for future intervention strategies.

While these strengths are notable, several limitations should be acknowledged: the observational study design precludes definitive causal conclusions despite rigorous adjustments for potential confounders. We could not adjust for the consumption of ultra‐processed foods (UPFs). Given that diets low in essential micronutrients often coexist with high UPF intake, residual confounding by UPFs may partially explain the observed associations. The analyses involving multiple vitamins and minerals were exploratory in nature, and no formal correction for multiple comparisons was applied. Some of the observed micronutrient associations may therefore reflect chance findings, and these results should be interpreted cautiously pending replication in independent cohorts. Although missing data accounted for approximately 10% of the original NHANES sample and survey weights were applied to maintain national representativeness, this approach may introduce a degree of selection bias if the missing data are not missing completely at random. Evaluating the CKM stage 0–3 population as a whole without stage‐stratified analyses limits our ability to provide stage‐specific dietary guidelines, which warrants cautious interpretation. Although we utilized the average of two separate dietary assessments to minimize the randomness of day‐to‐day fluctuations, our evaluation remains based on baseline habitual diet, and we lack continuous longitudinal data to track potential dietary modifications over subsequent years. Additionally, inherent differences in dietary assessment tools between the two cohorts precluded the complete harmonization of dietary indices, necessitating the use of different validated dietary patterns. Finally, while our findings are based on two large, representative Western cohorts, dietary habits and genetic susceptibilities vary significantly across populations. While our findings are based on two large, representative Western cohorts, dietary habits and genetic susceptibilities vary significantly across populations. Therefore, replication of these findings in diverse global cohorts, particularly in Asian, African, and Hispanic‐majority populations, is essential to confirm the universal applicability of these dietary recommendations for CKM syndrome. Nevertheless, the robust findings consistently demonstrate that plant‐based dietary patterns serve as an effective preventive approach against MACE in early‐stage CKM populations, providing compelling evidence for clinical practice and public health recommendations.

## 5 Conclusions

This observational study suggests that greater adherence to plant‐based dietary patterns (AMED and HPDI) and higher dietary intake of certain micronutrients, including carotenoids and key minerals, are associated with lower risks of MACE and mortality in individuals with CKM syndrome stages 0–3. These associations pertain to whole‐food sources; supplementation, particularly of iron and copper, should not be inferred from these findings and requires clinical guidance. Oxidative stress, phenotypic aging, and inflammatory markers may partially mediate these dietary associations, though causal conclusions cannot be drawn from observational data. Future intervention studies are needed to confirm these findings.

## Author Contributions

**Wen Gu:** conceptualization, investigation, formal analysis. **Kaixin Yuan:** conceptualization, writing – original draft, writing – review and editing, methodology, software. **Shuli Li:** conceptualization, project administration, resources. **Tian Wang:** conceptualization, data curation, supervision, formal analysis. **Yingqi Hou:** conceptualization, investigation, methodology, writing – original draft, writing – review and editing. **Yadan Xu:** conceptualization, methodology, software, formal analysis. **Jinjin Liu:** conceptualization, software, data curation. **Yanqiu Huang:** conceptualization, investigation, writing – original draft, data curation. **Wentao Shi:** conceptualization, writing – review and editing, writing – original draft, funding acquisition. **Tangyi Su:** conceptualization, validation, visualization. **Honglin Liu:** conceptualization, writing – original draft, writing – review and editing, funding acquisition. **Yangchen Lhamu:** conceptualization, investigation, validation.

## Funding

This study was supported by grants from the Medical Scientific Research Foundation of Guangdong Province of China (Grant No. A2026121), Zhongshan Science and Technology Bureau Project (No. 2025B1020), Shanghai Ninth Hospital Management Research Project, and Science and Technology Research Project of the Education Department of Jilin Province (2024 Key Project).

## Ethics Statement

The UK Biobank received ethical approval from the North West Multi‐center Research Ethics Committee (Approved Research ID: 194423, Approval date: October 30, 2024). All participants gave written informed consent before enrollment in the study, which was conducted in accordance with the principles of the Declaration of Helsinki. The National Center for Health Statistics and Ethics Review Board approved the protocol for NHANES.

## Consent

All participants provided written informed consent.

## Conflicts of Interest

The authors declare no conflicts of interest.

## Supporting information


**Figure S1:** Flow chart of UK Biobank.

**Figure S2:** Flow chart of NHANES.

**Figure S3:** Association of diets and minerals with CVD mortality in the UK Biobank.

**Figure S4:** Association of diets and minerals with myocardial infarction in the UK Biobank.

**Figure S5:** Association of diets and minerals with stroke in the UK Biobank.

**Figure S6:** Association of vitamins with CVD mortality in the UK Biobank.

**Figure S7:** Association of vitamins with myocardial infarction in the UK Biobank.

**Figure S8:** Association of vitamins with stroke in the UK Biobank.

**Table S1:** Food composition of dietary pattern scores.

**Table S2:** Association of AMED and HPDI with MACE in sex subgroups based on participants with CKM stages 0–3 from UK Biobank.

**Table S3:** Association of AMED and HPDI with all‐cause mortality in sex subgroups based on participants with CKM stages 0–3 from UK Biobank.

**Table S4:** Association of AMED and HPDI with CVD mortality in sex subgroups based on participants with CKM stages 0–3 from UK Biobank.

**Table S5:** Association of AMED and HPDI with stroke in sex subgroups based on participants with CKM stages 0–3 from UK Biobank.

**Table S6:** Association of AMED and HPDI with myocardial infarction in sex subgroups based on participants with CKM stages 0–3 from UK Biobank.

**Table S7:** Association of AMED and DII with all‐cause mortality in sex subgroups based on participants with CKM stages 0–3 from NHANES.

**Table S8:** Association of AMED and DII with CVD mortality in sex subgroups based on participants with CKM stages 0–3 from NHANES.

## Data Availability

The UK Biobank data are available to approved researchers through a formal application process via the UK Biobank Access Management System (https://www.ukbiobank.ac.uk), and the NHANES data are publicly available and freely accessible through the National Center for Health Statistics website (https://www.cdc.gov/nchs/nhanes). The datasets that were used and evaluated in this study can be obtained from the corresponding author upon making a reasonable request.
