# Supplementary figures and images for: Associations of Dietary Patterns and Micronutrients With Major Adverse Cardiovascular Events and Mortality Among Populations With Cardiovascular‐Kidney‐Metabolic Syndrome Stages 0–3: Results From Two Prospective Cohorts
Source: Food Sci Nutr. 2026 Jul 2;14(7):e72082. doi: 10.1002/fsn3.72082 (PMC13326665; doi:10.1002/fsn3.72082)

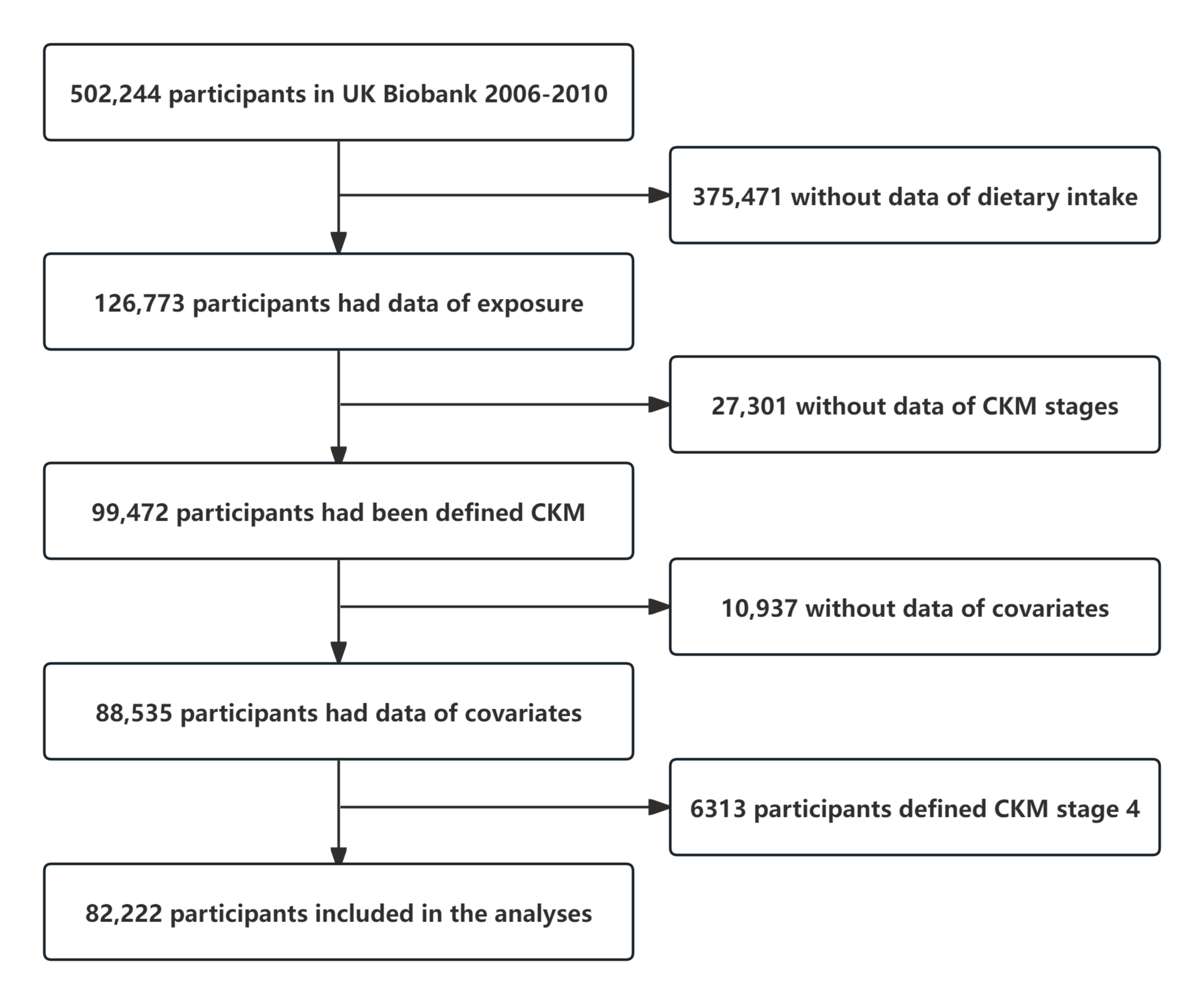

Supplement: Supplementary file 1 — Figure S1: Flow chart of UK Biobank. [file FSN3-14-e72082-s016.tif]

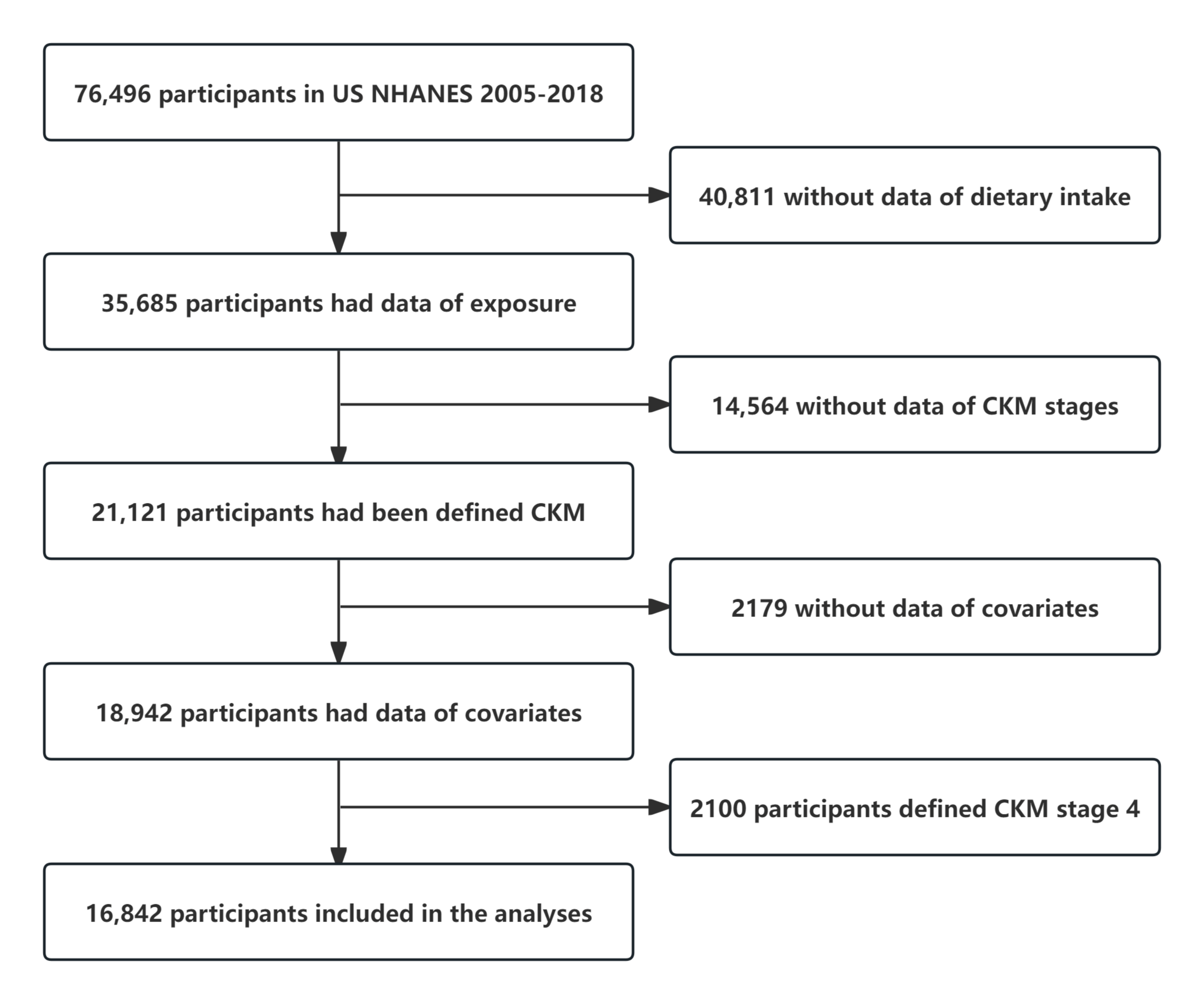

Supplement: Supplementary file 2 — Figure S2: Flow chart of NHANES. [file FSN3-14-e72082-s002.tif]

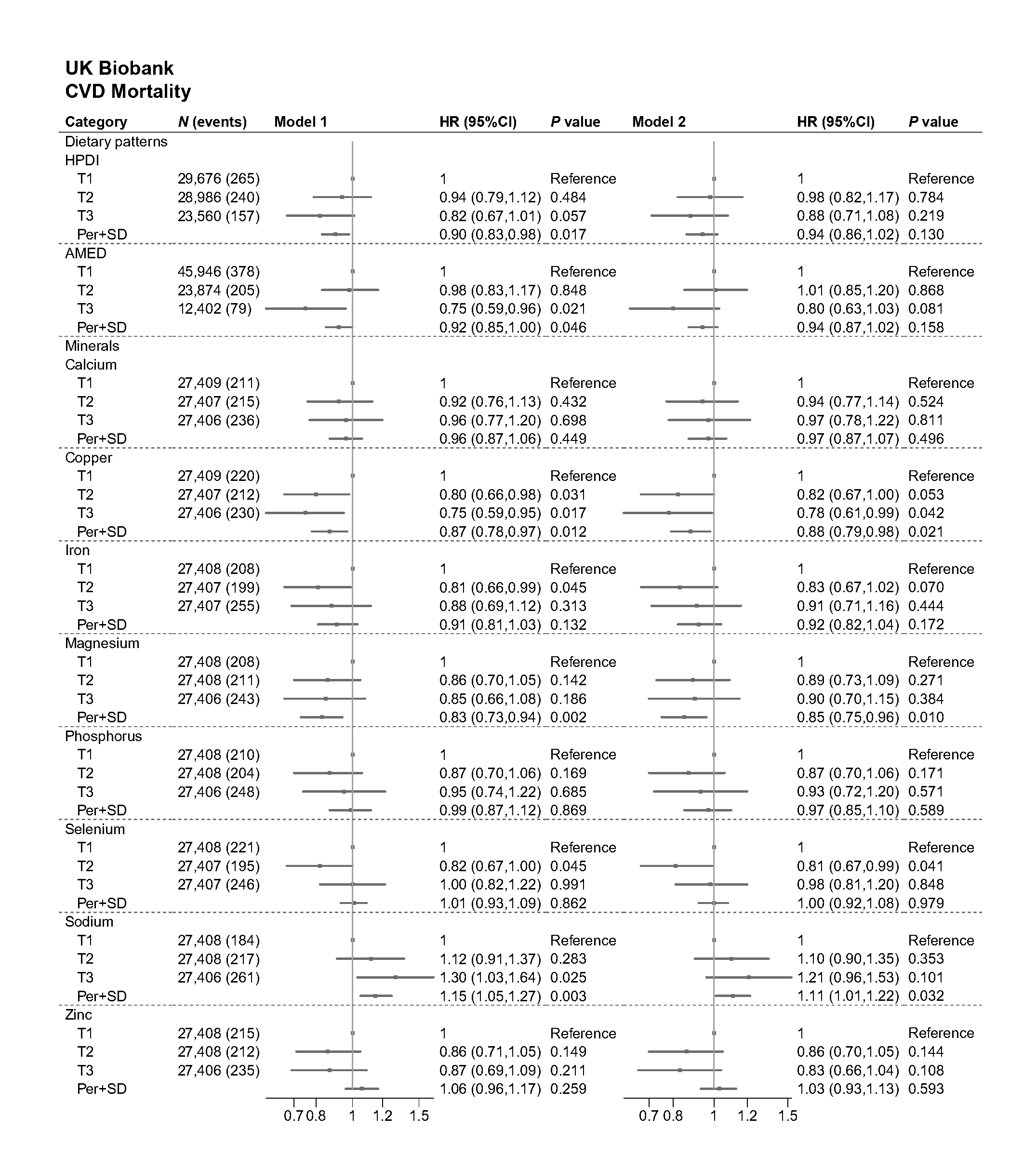

Supplement: Supplementary file 3 — Figure S3: Association of diets and minerals with CVD mortality in the UK Biobank. [file FSN3-14-e72082-s015.tif]

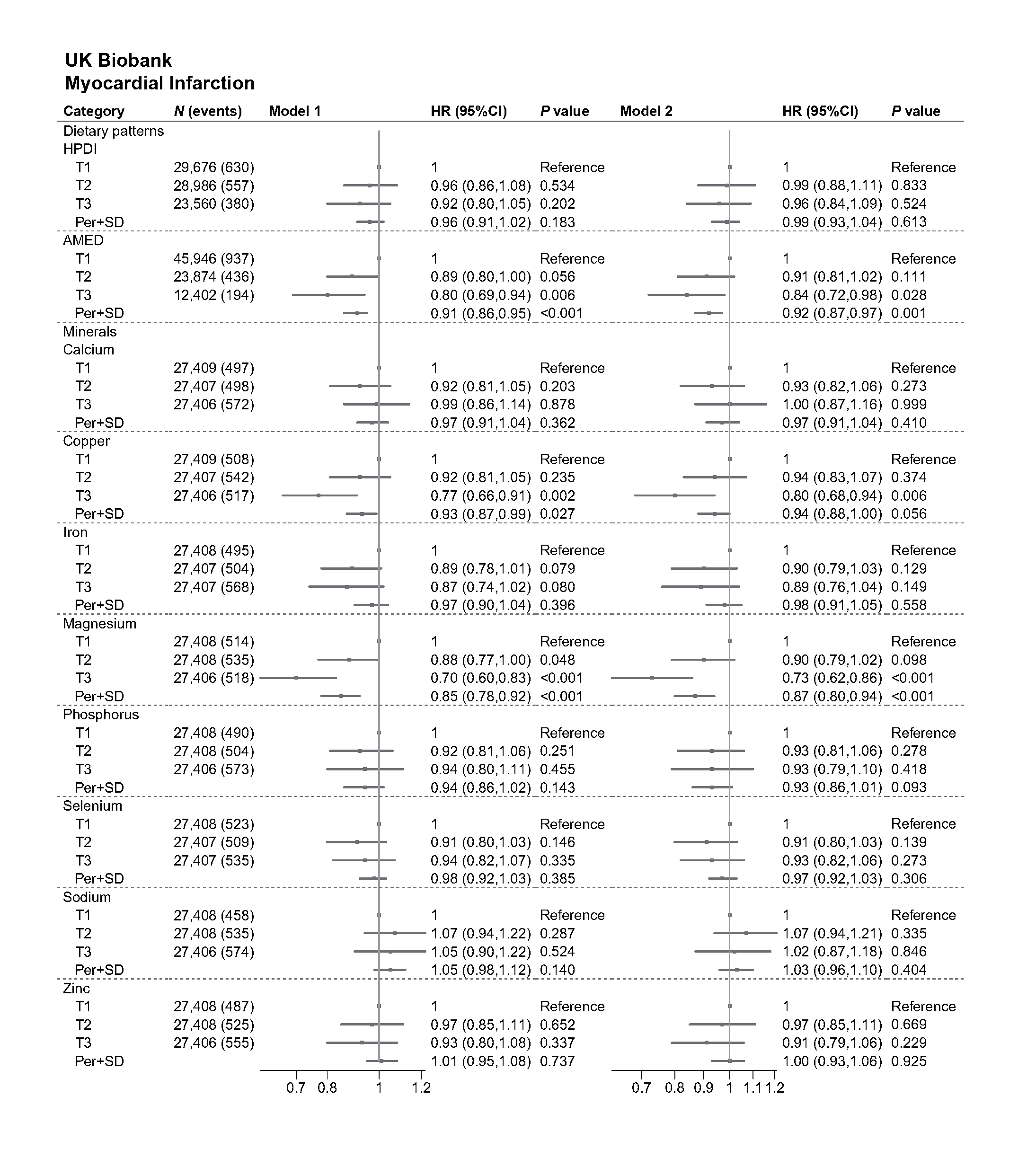

Supplement: Supplementary file 4 — Figure S4: Association of diets and minerals with myocardial infarction in the UK Biobank. [file FSN3-14-e72082-s003.tif]

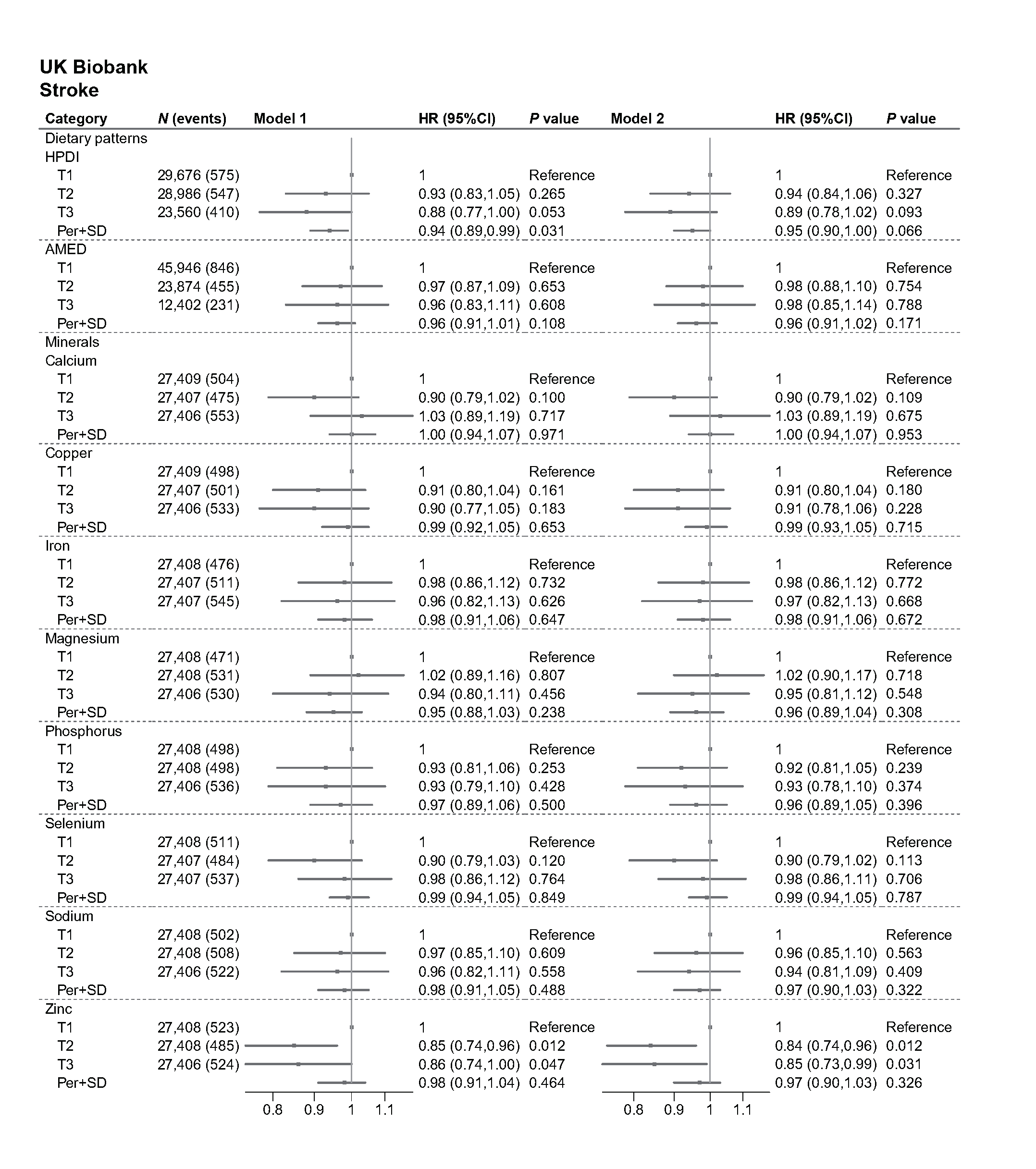

Supplement: Supplementary file 5 — Figure S5: Association of diets and minerals with stroke in the UK Biobank. [file FSN3-14-e72082-s014.tif]

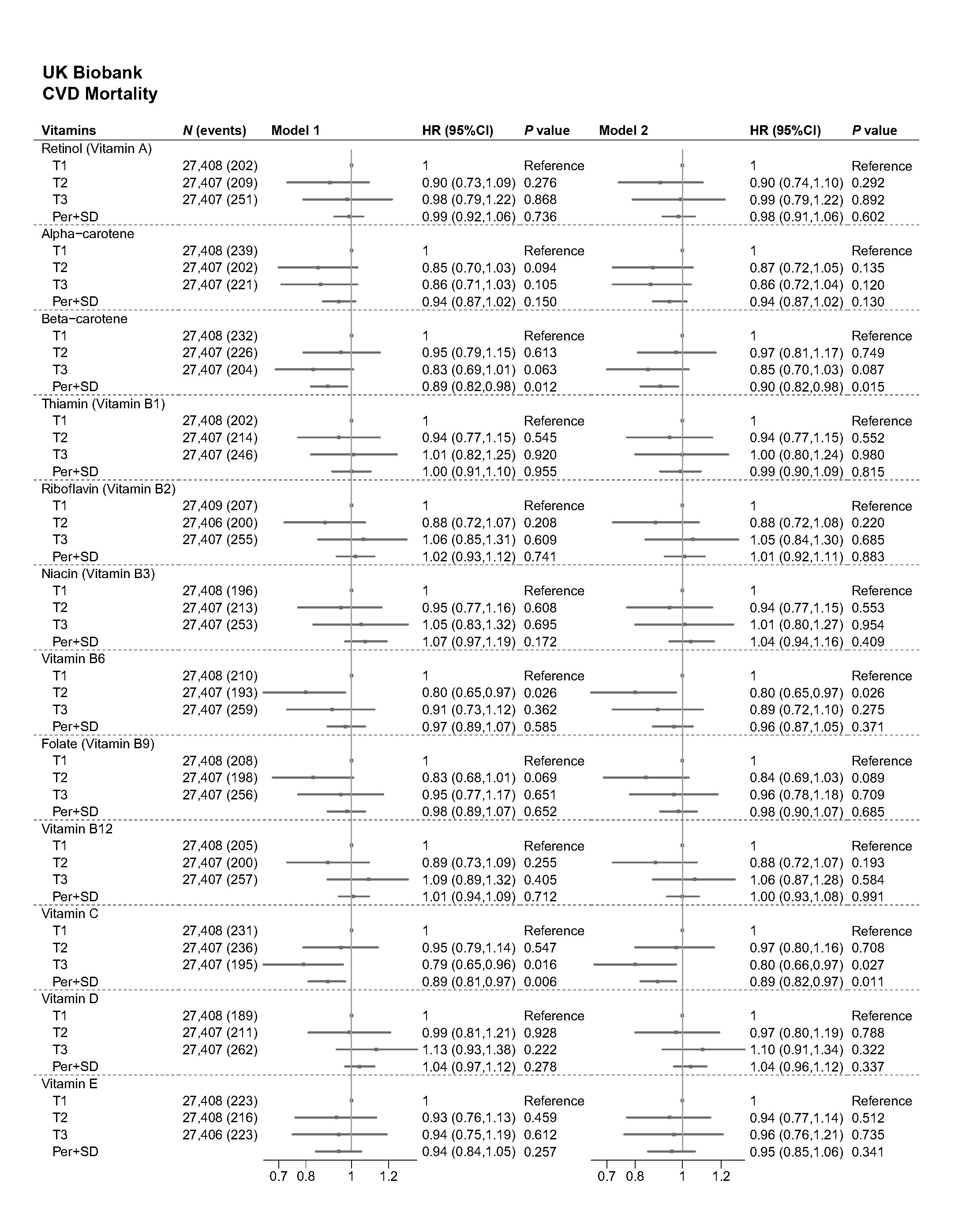

Supplement: Supplementary file 6 — Figure S6: Association of vitamins with CVD mortality in the UK Biobank. [file FSN3-14-e72082-s012.tif]

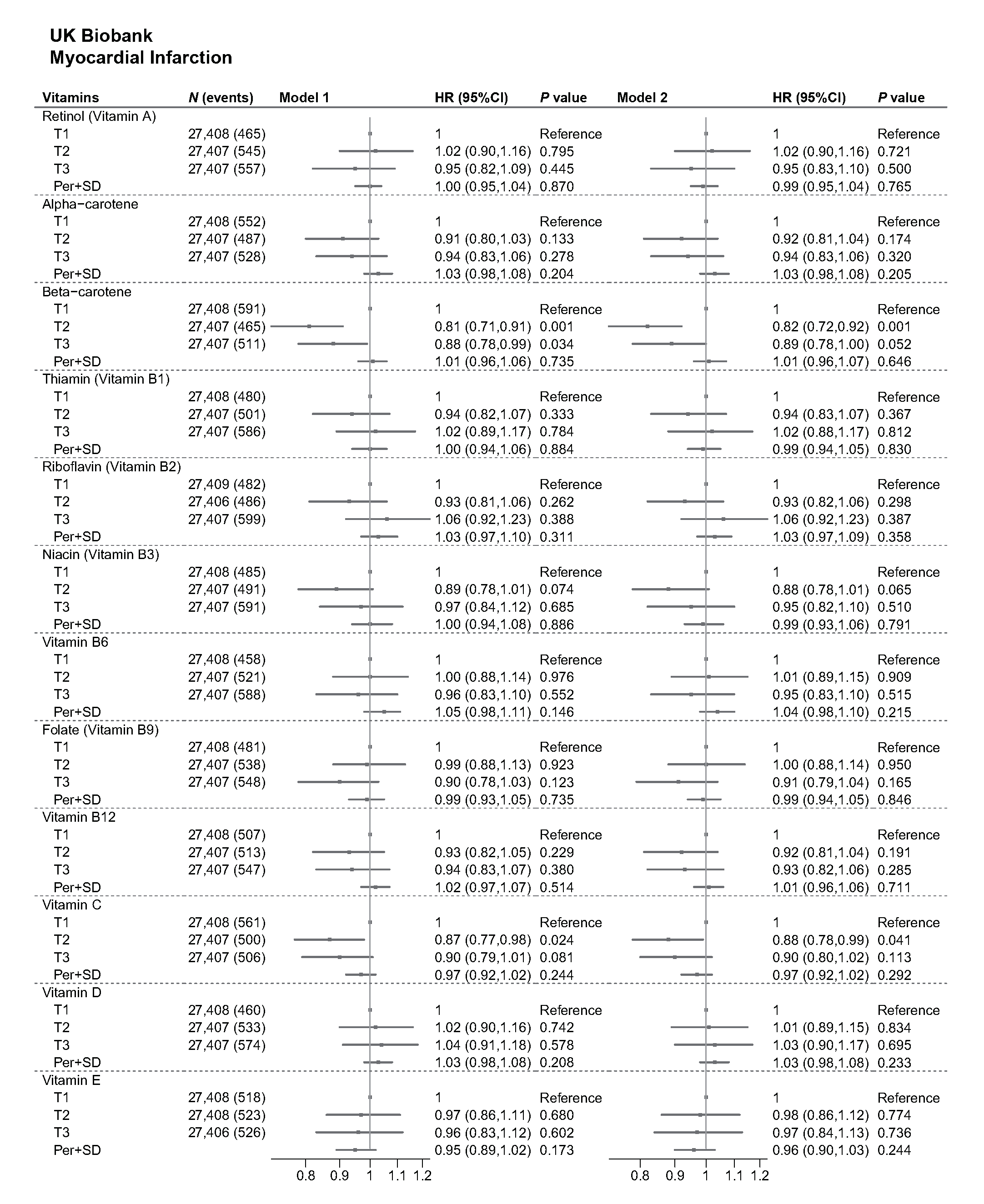

Supplement: Supplementary file 7 — Figure S7: Association of vitamins with myocardial infarction in the UK Biobank. [file FSN3-14-e72082-s005.tif]

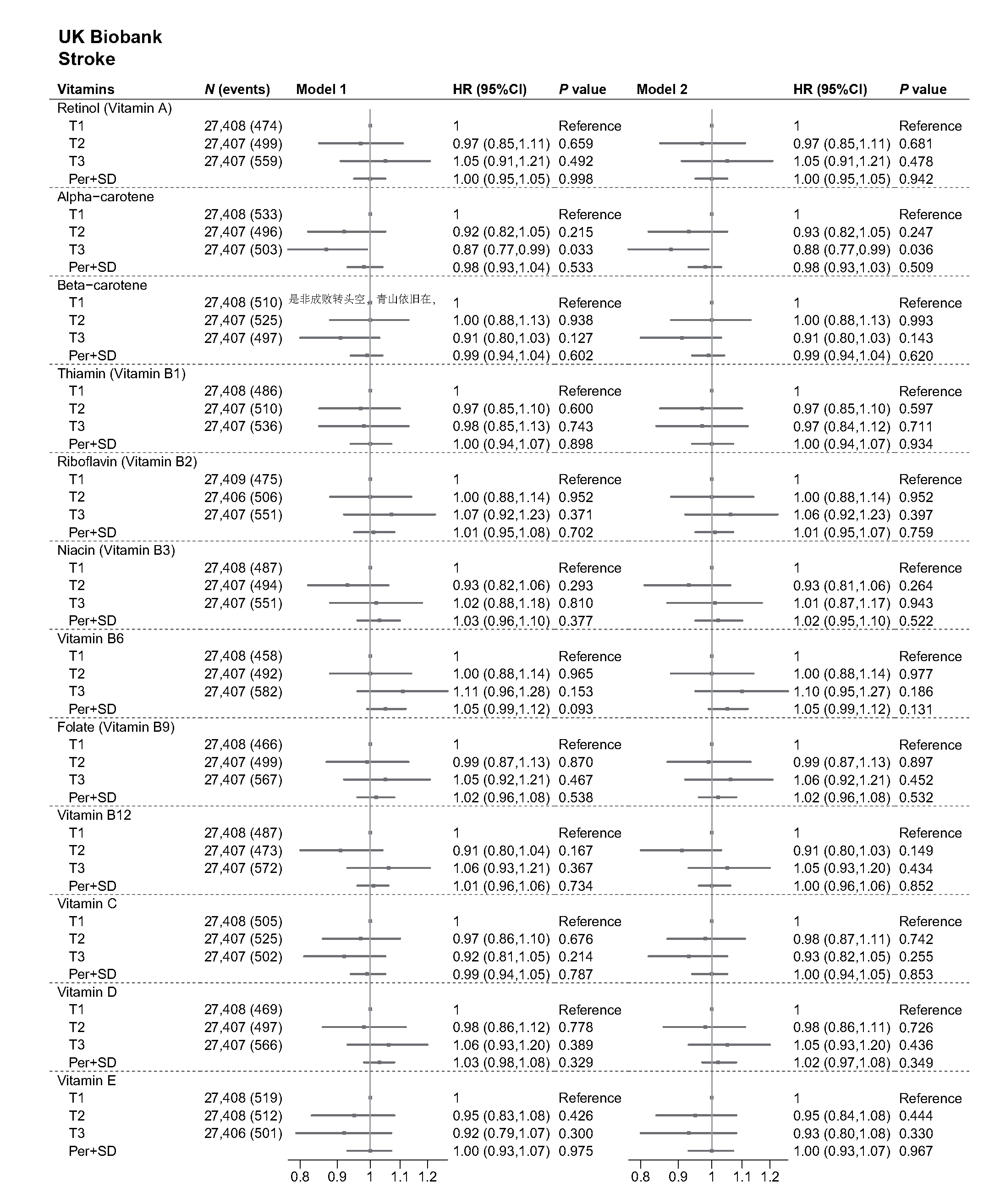

Supplement: Supplementary file 8 — Figure S8: Association of vitamins with stroke in the UK Biobank. [file FSN3-14-e72082-s010.tif]
