# Supplementary material for: Associations of Dietary Patterns and Micronutrients With Major Adverse Cardiovascular Events and Mortality Among Populations With Cardiovascular‐Kidney‐Metabolic Syndrome Stages 0–3: Results From Two Prospective Cohorts
Source: Food Sci Nutr. 2026 Jul 2;14(7):e72082. doi: 10.1002/fsn3.72082 (PMC13326665; doi:10.1002/fsn3.72082)
Supplement: Supplementary file 9 — Table S1: Food composition of dietary pattern scores. [file FSN3-14-e72082-s004.docx]

**Table S1.** Food Composition of Dietary Pattern Scores.

| Dietary Pattern | Food Composition (maximum score) | Standard for score |
| --- | --- | --- |
| AMED(1) | **Beneficial dietary components:**  vegetables, legumes, fruits, nuts, whole grains, fish,  **Detrimental dietary components:**  meats, red and processed alcohol, and a monounsaturated/saturated fat ratio | Altogether, nine food groups were constructed as index dietary components, namely vegetables, fruits and nuts, cereals, legumes, dairy products, fish and seafood, meat, alcohol, and monounsaturated-to-saturated fat (M/S) ratio. For dietary components that are presumed to be beneficial (i.e., vegetables, fruits and nuts, cereals, legumes, fish and seafood, and a high M/S ratio), we scored a woman that consumed below the median level of the entire cohort as “0” and a woman that consumed at or above the cohort median as 1. For dietary components that are presumed to be less beneficial (i.e., dairy and meat products), a consumption level below the cohort median was given a score of 1 whereas a consumption level at or above the cohort median was given a score of 0. A moderate level of alcohol consumption (5-25 g/day) was scored 1, or 0 otherwise. Scores on all nine components were then summed up as a proxy for adherence to MDP, with the value 0 as the minimal and 9 as the maximal adherence. |
| HPDI(2) | **Plant-based food groups (Positive):**  whole grains, fruits, vegetables, nuts, legumes, tea and/or coffee  **Animal-based food groups (Reverse):**  animal fat, dairy, eggs, fish/seafood, meat, miscellaneous animal-based foods  **Other food groups (Reverse):**  refined grains, potatoes, sugary drinks, fruit juices, sweets and/or desserts | \| Food groups \| Food items \| Scoring \| \| --- \| --- \| --- \| \| Whole grains \| Porridge, muesli, oat crunch, bran cereal, cereal bar, non-white bread (flour types, brown, wholemeal, other type), seeded or other bread, crispbread, whole-wheat cereal, other cereal, whole meal pasta, brown rice, couscous, other cooked grains \| Positive \| \| Fruits \| Mixed fruit, apple, banana, berries, cherries, grapefruit, grapes, mango, melon, orange, orange-like small fruits, peach/nectarine, pear, pineapple, plum, other fruits, stewed/cooked fruit, prunes, other dried fruit \| Positive \| \| Vegetables \| Mixed vegetables, vegetable pieces, coleslaw, side salad, beetroot, broccoli, butternut squash, cabbage/kale, carrots, cauliflower, celery, courgette, cucumber, garlic, leeks, lettuce, mushrooms, onion, parsnip, sweet peppers, spinach, sprouts, sweetcorn, sweet potato, fresh tomatoes, cooked or tinned tomatoes, turnip/swede, watercress, other vegetable \| Positive \| \| Nuts \| Salted peanuts, unsalted peanuts, salted nuts, unsalted nuts, seeds \| Positive \| \| Legumes \| Baked beans, other beans or lentils, broad beans, green beans, peas, soy or vegetable milk, vegetarian sausages/burgers, tofu, quorn, other vegetarian alternative \| Positive \| \| Tea and coffee \| Instant coffee, filtered coffee, cappuccino, latte, espresso, other coffee drinks, standard tea, rooibos tea, green tea, herbal tea, other tea \| Positive \| \| Refined grains \| Sweetened cereal, plain cereal, white bread, naan bread, garlic bread, white pasta, white rice, pancake, scotch pancake, croissant, scone, savoury or cheesy biscuits, other savoury snack, snackpot \| Reverse \| \| Potatoes \| Fried potatoes, boiled/baked potatoes, mashed potatoes, crisps (e.g., potato chips) \| Reverse \| \| Sugary drinks \| Low calorie or diet drinks (e.g. fizzy, squash), carbonated (fizzy) drinks, squash or cordial \| Reverse \| \| Fruit juices \| Orange juice, grapefruit juice, other fruit/vegetable juice, fruit smoothie \| Reverse \| \| Sweets and desserts \| Double crust pie, single crust pie/flan, crumble topping, Yorkshire pudding, Danish pastry, fruitcake, cake, doughnuts, sponge pudding, other dessert, chocolate bar, white chocolate, milk chocolate, dark chocolate, chocolate-covered raisin, chocolate sweet, diet sweets, chocolate-covered biscuits, chocolate biscuits, sweet biscuits, other sweets \| Reverse \| \| Animal fat \| Butter on bread/crackers (spreadable, low fat, normal fat, or unknown type), dairy spread on bread/crackers (very low fat, low fat, normal fat, unknown type) \| Reverse \| \| Dairy \| Milk, dairy smoothie, flavored milk, yogurt, ice-cream, low fat hard cheese, hard cheese, soft cheese, blue cheese, low fat cheese spread, cheese spread, cottage cheese, feta cheese, mozzarella cheese, goat's cheese, other cheese, cheesecake, milk-based pudding, other milk-based pudding \| Reverse \| \| Eggs \| Whole eggs, omelettes or scrambled egg, eggs in sandwiches, scotch egg, other egg dishes \| Reverse \| \| Fish/seafood \| Tinned tuna, oily fish, breaded fish, battered fish, white fish, prawns, lobster/crab,  shellfish, other fish \| Reverse \| \| Meat \| Sausage, beef, pork, lamb, crumbed or deep-fried poultry, poultry, bacon, ham, liver, other meat \| Reverse \| \| Miscellaneous animal based foods \| Pizza, Indian snacks \| Reverse \| |
| DII(3) | **Pro-inflammatory Components:**  saturated fatty acids, total fat, energy, cholesterol.  **Anti-inflammatory Components:**  polyunsaturated fatty acids, n-3 fatty acids, n-6 fatty acids, monounsaturated fatty acids, fiber, alcohol, vitamin(V) A, VC, VD, VE, magnesium, zinc, selenium, folate, carotene, caffeine.  **Neutral or Context-Dependent Components:**  protein, carbohydrates, niacin, thiamine, VB2, VB6, VB12, iron. | Calculation of the dietary inflammatory index Calculation of the DII is based on dietary intake data that are then linked to the regionally representative world database that provided a robust estimate of a mean and standard deviation for each parameter. These then become the multipliers to express an individual’s exposure relative to the ‘standard global mean’ as a Z-score. This is achieved by subtracting the ‘standard mean’ from the amount reported and dividing this value by its standard deviation. To minimize the effect of ‘right skewing’, this value is converted to a percentile score. To achieve a symmetrical distribution with values centred on 0 (null) and bounded between -1 (maximally anti-inflammatory) and +1 (maximally pro-inflammatory), each percentile score is doubled and then ‘1’ is subtracted. The centred percentile value for each food parameter is then multiplied by its respective ‘overall food parameter-specific inflammatory effect score’ to obtain the ‘food parameter-specific DII score’. Finally, all of the ‘food parameter-specific DII scores’ are summed to create the ‘overall DII score’ for an individual. This approach both ‘anchors’ the individual’s exposure to a robust range of dietary patterns in a variety of cultural traditions and obviates completely the problem of non-comparability of units because the Z-scores and percentiles are independent of the units of measurement. |

Note: All scores were calculated by R package Dietaryindex, details can be found in references(4).

Reference:

1. W Y, M L, R C, Cm H, F F, S S. Mediterranean diet and depression: a population-based cohort study. Int J Behav Nutr Phys Act [Internet]. 2021 Nov 27 [cited 2024 Sep 8];18(1). Available from: http://pubmed-ncbi-nlm-nih-gov-s.webvpn.njmu.edu.cn:8118/34838037/

2. Shang X, Liu J, Zhu Z, Zhang X, Huang Y, Liu S, et al. Healthy dietary patterns and the risk of individual chronic diseases in community-dwelling adults. Nat Commun. 2023 Oct 23;14(1):6704.

3. N S, Se S, Tg H, Jr H, Jr H. Designing and developing a literature-derived, population-based dietary inflammatory index. Public Health Nutr [Internet]. 2014 Aug [cited 2025 Apr 25];17(8). Available from: http://pubmed-ncbi-nlm-nih-gov-s.webvpn.njmu.edu.cn:8118/23941862/

4. Jj Z, Ra H, Al D, Mm L, L B, D L, et al. Dietaryindex: a user-friendly and versatile R package for standardizing dietary pattern analysis in epidemiological and clinical studies. Am J Clin Nutr [Internet]. 2024 Nov [cited 2025 Apr 25];120(5). Available from: http://pubmed-ncbi-nlm-nih-gov-s.webvpn.njmu.edu.cn:8118/39182618/
