# Supplementary material for: Associations of Dietary Patterns and Micronutrients With Major Adverse Cardiovascular Events and Mortality Among Populations With Cardiovascular‐Kidney‐Metabolic Syndrome Stages 0–3: Results From Two Prospective Cohorts
Source: Food Sci Nutr. 2026 Jul 2;14(7):e72082. doi: 10.1002/fsn3.72082 (PMC13326665; doi:10.1002/fsn3.72082)
Supplement: Supplementary file 11 — Table S3: Association of AMED and HPDI with all‐cause mortality in sex subgroups based on participants with CKM stages 0–3 from UK Biobank. [file FSN3-14-e72082-s008.docx]

**Table S3.** Association of AMED and HPDI with all-cause mortality in sex subgroups based on participants with CKM stages 0-3 from UK Biobank.

| **Dietary patterns** |  | **All-cause Mortality** | | | | | | | | |  |
| --- | --- | --- | --- | --- | --- | --- | --- | --- | --- | --- | --- |
|  | **N (events)** | **Model 1** | | | **Model 2** | | | **Model 3** | | | **P interaction** |
|  |  | **HR** | **95%CI** | **P-value** | **HR** | **95%CI** | **P-value** | **HR** | **95%CI** | **P-value** |  |
| **Male** |  |  |  |  |  |  |  |  |  |  |  |
| **AMED** |  |  |  |  |  |  |  |  |  |  | 0.330 |
| T1 | 12,686 (5482) | 1 | 1 | Reference | 1 | 1 | Reference | 1 | 1 | Reference |  |
| T2 | 12,513 (5102) | 0.84 | (0.77,0.93) | <0.001 | 0.86 | (0.79,0.95) | 0.002 | 0.88 | (0.80,0.96) | 0.007 |  |
| T3 | 11,430 (4325) | 0.75 | (0.66,0.85) | <0.001 | 0.77 | (0.67,0.88) | <0.001 | 0.80 | (0.70,0.91) | 0.001 |  |
| Per+SD |  | 0.88 | (0.84,0.91) | <0.001 | 0.89 | (0.85,0.93) | <0.001 | 0.90 | (0.86,0.94) | <0.001 |  |
| **HPDI** |  |  |  |  |  |  |  |  |  |  | 0.759 |
| T1 | 12,695 (788) | 1 | 1 | Reference | 1 | 1 | Reference | 1 | 1 | Reference |  |
| T2 | 13,590 (873) | 0.91 | (0.82,1.00) | 0.048 | 0.92 | (0.83,1.01) | 0.081 | 0.94 | (0.85,1.04) | 0.207 |  |
| T3 | 11,028 (691) | 0.84 | (0.75,0.93) | 0.001 | 0.85 | (0.76,0.94) | 0.002 | 0.88 | (0.79,0.98) | 0.017 |  |
| Per+SD |  | 0.91 | (0.87,0.95) | <0.001 | 0.91 | (0.87,0.95) | <0.001 | 0.93 | (0.89,0.97) | 0.001 |  |
| **Female** |  |  |  |  |  |  |  |  |  |  |  |
| **AMED** |  |  |  |  |  |  |  |  |  |  |  |
| T1 | 23,457 (899) | 1 | 1 | Reference | 1 | 1 | Reference | 1 | 1 | Reference |  |
| T2 | 13,709 (515) | 0.89 | (0.80,0.99) | 0.033 | 0.9 | (0.81,1.01) | 0.069 | 0.92 | (0.82,1.03) | 0.131 |  |
| T3 | 7743 (268) | 0.82 | (0.72,0.94) | 0.005 | 0.84 | (0.73,0.96) | 0.011 | 0.87 | (0.76,1.00) | 0.047 |  |
| Per+SD |  | 0.91 | (0.87,0.96) | <0.001 | 0.92 | (0.88,0.97) | 0.001 | 0.94 | (0.89,0.98) | 0.009 |  |
| **HPDI** |  |  |  |  |  |  |  |  |  |  |  |
| T1 | 17,465 (662) | 1 | 1 | Reference | 1 | 1 | Reference | 1 | 1 | Reference |  |
| T2 | 13,795 (529) | 0.89 | (0.79,0.99) | 0.038 | 0.90 | (0.80,1.01) | 0.068 | 0.92 | (0.82,1.03) | 0.140 |  |
| T3 | 13,649 (491) | 0.79 | (0.70,0.89) | <0.001 | 0.80 | (0.71,0.91) | 0.000 | 0.83 | (0.74,0.94) | 0.003 |  |
| Per+SD |  | 0.89 | (0.84,0.93) | <0.001 | 0.89 | (0.85,0.94) | <0.001 | 0.91 | (0.87,0.96) | <0.001 |  |

**Note:**

Model 1: age (continuous), ethnicity/race (White, Asian or Asian British, Black or Black British, Chinese, Mixed, Other ethnic group), total energy intake (continuous).

Model 2: age (continuous), ethnicity/race (White, Asian or Asian British, Black or Black British, Chinese, Mixed, Other ethnic group), educational level (less than high school, high school and above), Townsend deprivation index (T1, T2, T3), smoking status (Yes or No), alcohol consumption (continuous), physical activity (adequate, inadequate), total energy intake (continuous).

Model 3: age (continuous), ethnicity/race (White, Asian or Asian British, Black or Black British, Chinese, Mixed, Other ethnic group), educational level (less than high school, high school and above), Townsend deprivation index (T1, T2, T3), smoking status (Yes or No), alcohol consumption (continuous), physical activity (adequate, inadequate), BMI (continuous), history of diabetes (Yes or No), total energy intake (continuous).

1. values less than 0.05 (p < 0.05) were considered significant.

**Abbreviations:** CKM=Cardiovascular-Kidney-Metabolic Syndrome, T=tertile, SD=standard deviation, BMI=body mass index, AMED=Alternate Mediterranean Diet, HPDI=Healthful Plant-Based Diet Index, HR=hazard ratio, CI=confidence interval, N=number.
