# Supplementary material for: Associations of Dietary Patterns and Micronutrients With Major Adverse Cardiovascular Events and Mortality Among Populations With Cardiovascular‐Kidney‐Metabolic Syndrome Stages 0–3: Results From Two Prospective Cohorts
Source: Food Sci Nutr. 2026 Jul 2;14(7):e72082. doi: 10.1002/fsn3.72082 (PMC13326665; doi:10.1002/fsn3.72082)
Supplement: Supplementary file 15 — Table S7: Association of AMED and DII with all‐cause mortality in sex subgroups based on participants with CKM stages 0–3 from NHANES. [file FSN3-14-e72082-s001.docx]

**Table S7.** Association of AMED and DII with all-cause mortality in sex subgroups based on participants with CKM stages 0-3 from NHANES.

| **Dietary patterns** |  | **All-cause Mortality** | | | | | | | | |  |
| --- | --- | --- | --- | --- | --- | --- | --- | --- | --- | --- | --- |
|  | **N (events)** | **Model 1** | | | **Model 2** | | | **Model 3** | | | **P interaction** |
|  |  | **HR** | **95%CI** | **P-value** | **HR** | **95%CI** | **P-value** | **HR** | **95%CI** | **P-value** |  |
| **Male** |  |  |  |  |  |  |  |  |  |  |  |
| **AMED** |  |  |  |  |  |  |  |  |  |  | 0.998 |
| T1 | 3183 (260) | 1 | 1 | Reference | 1 | 1 | Reference | 1 | 1 | Reference |  |
| T2 | 2830 (283) | 1 | (0.84,1.18) | 0.955 | 1.14 | (0.96,1.35) | 0.137 | 1.14 | (0.96,1.35) | 0.140 |  |
| T3 | 1979 (199) | 0.69 | (0.57,0.83) | <0.001 | 0.84 | (0.69,1.02) | 0.075 | 0.83 | (0.68,1.00) | 0.054 |  |
| Per+SD |  | 0.85 | (0.79,0.91) | <0.001 | 0.92 | (0.86,0.99) | 0.033 | 0.92 | (0.85,0.99) | 0.021 |  |
| **DII** |  |  |  |  |  |  |  |  |  |  | 0.926 |
| T1 | 2664 (189) | 1 | 1 | Reference | 1 | 1 | Reference | 1 | 1 | Reference |  |
| T2 | 2664 (240) | 1.37 | (1.12,1.67) | 0.002 | 1.25 | (1.03,1.53) | 0.025 | 1.25 | (1.03,1.53) | 0.026 |  |
| T3 | 2664 (313) | 1.72 | (1.39,2.13) | <0.001 | 1.33 | (1.06,1.66) | 0.013 | 1.35 | (1.08,1.69) | 0.008 |  |
| Per+SD |  | 1.31 | (1.19,1.43) | <0.001 | 1.16 | (1.06,1.28) | 0.002 | 1.18 | (1.07,1.30) | 0.001 |  |
| **Female** |  |  |  |  |  |  |  |  |  |  |  |
| **AMED** |  |  |  |  |  |  |  |  |  |  |  |
| T1 | 4552 (296) | 1 | 1 | Reference | 1 | 1 | Reference | 1 | 1 | Reference |  |
| T2 | 1510 (86) | 0.59 | (0.47,0.76) | <0.001 | 0.67 | (0.52,0.85) | 0.001 | 0.67 | (0.52,0.85) | 0.001 |  |
| T3 | 2788 (168) | 0.56 | (0.47,0.68) | <0.001 | 0.68 | (0.56,0.83) | <0.001 | 0.68 | (0.56,0.83) | <0.001 |  |
| Per+SD |  | 0.75 | (0.69,0.82) | <0.001 | 0.83 | (0.76,0.90) | <0.001 | 0.83 | (0.75,0.90) | <0.001 |  |
| **DII** |  |  |  |  |  |  |  |  |  |  |  |
| T1 | 2950 (148) | 1 | 1 | Reference | 1 | 1 | Reference | 1 | 1 | Reference |  |
| T2 | 2950 (175) | 1.37 | (1.09,1.72) | 0.007 | 1.23 | (0.97,1.55) | 0.085 | 1.24 | (0.98,1.56) | 0.074 |  |
| T3 | 2950 (227) | 1.73 | (1.35,2.22) | <0.001 | 1.43 | (1.11,1.84) | 0.006 | 1.46 | (1.13,1.88) | 0.004 |  |
| Per+SD |  | 1.29 | (1.16,1.44) | <0.001 | 1.17 | (1.05,1.30) | 0.006 | 1.17 | (1.05,1.31) | 0.004 |  |

**Note:**

Model 1: age (continuous), ethnicity/race (Mexican American, Other Hispanic, Non-Hispanic White, Non-Hispanic Black, Other Race - Including Multi-Racial), total energy intake (continuous).

Model 2: age (continuous), ethnicity/race (Mexican American, Other Hispanic, Non-Hispanic White, Non-Hispanic Black, Other Race - Including Multi-Racial), educational level (less than high school, high school and above), ratio of family income to poverty (< 1.3, 1.3-3.5, ≥ 3.5), smoking status (Yes or No), alcohol consumption (continuous), physical activity (adequate, inadequate), total energy intake (continuous).

Model 3: age (continuous), ethnicity/race (Mexican American, Other Hispanic, Non-Hispanic White, Non-Hispanic Black, Other Race - Including Multi-Racial), educational level (less than high school, high school and above), ratio of family income to poverty (< 1.3, 1.3-3.5, ≥ 3.5), smoking status (Yes or No), alcohol consumption (continuous), physical activity (adequate, inadequate), BMI (continuous), history of diabetes (Yes or No), total energy intake (continuous).

1. values less than 0.05 (p < 0.05) were considered significant.

**Abbreviations:** CKM=Cardiovascular-Kidney-Metabolic Syndrome, NHANES=National Health and Nutrition Examination Survey, T=tertile, SD=standard deviation, BMI=body mass index, AMED=Alternate Mediterranean Diet, DII=Dietary Inflammation Index, HR=hazard ratio, CI=confidence interval, N=number.
