# Supplementary material for: Associations of Dietary Patterns and Micronutrients With Major Adverse Cardiovascular Events and Mortality Among Populations With Cardiovascular‐Kidney‐Metabolic Syndrome Stages 0–3: Results From Two Prospective Cohorts
Source: Food Sci Nutr. 2026 Jul 2;14(7):e72082. doi: 10.1002/fsn3.72082 (PMC13326665; doi:10.1002/fsn3.72082)
Supplement: Supplementary file 16 — Table S8: Association of AMED and DII with CVD mortality in sex subgroups based on participants with CKM stages 0–3 from NHANES. [file FSN3-14-e72082-s009.docx]

**Table S8.** Association of AMED and DII with CVD mortality in sex subgroups based on participants with CKM stages 0-3 from NHANES.

| **Dietary patterns** |  | **CVD Mortality** | | | | | | | | |  |
| --- | --- | --- | --- | --- | --- | --- | --- | --- | --- | --- | --- |
|  | **N (events)** | **Model 1** | | | **Model 2** | | | **Model 3** | | | **P interaction** |
|  |  | **HR** | **95%CI** | **P-value** | **HR** | **95%CI** | **P-value** | **HR** | **95%CI** | **P-value** |  |
| **Male** |  |  |  |  |  |  |  |  |  |  |  |
| **AMED** |  |  |  |  |  |  |  |  |  |  | 0.124 |
| T1 | 3183 (56) | 1 | 1 | Reference | 1 | 1 | Reference | 1 | 1 | Reference |  |
| T2 | 2830 (64) | 1.03 | (0.72,1.48) | 0.875 | 1.14 | (0.79,1.65) | 0.474 | 1.15 | (0.80,1.65) | 0.455 |  |
| T3 | 1979 (59) | 0.88 | (0.61,1.27) | 0.497 | 1.01 | (0.69,1.48) | 0.949 | 1.03 | (0.70,1.51) | 0.883 |  |
| Per+SD |  | 0.91 | (0.79,1.04) | 0.170 | 0.96 | (0.82,1.11) | 0.556 | 0.96 | (0.83,1.12) | 0.614 |  |
| **DII** |  |  |  |  |  |  |  |  |  |  | 0.478 |
| T1 | 2664 (46) | 1 | 1 | Reference | 1 | 1 | Reference | 1 | 1 | Reference |  |
| T2 | 2664 (55) | 1.28 | (0.86,1.92) | 0.224 | 1.22 | (0.81,1.83) | 0.337 | 1.2 | (0.80,1.81) | 0.376 |  |
| T3 | 2664 (78) | 1.72 | (1.12,2.65) | 0.014 | 1.42 | (0.91,2.23) | 0.124 | 1.43 | (0.91,2.24) | 0.122 |  |
| Per+SD |  | 1.28 | (1.06,1.54) | 0.009 | 1.18 | (0.97,1.42) | 0.095 | 1.18 | (0.97,1.43) | 0.096 |  |
| **Female** |  |  |  |  |  |  |  |  |  |  |  |
| **AMED** |  |  |  |  |  |  |  |  |  |  |  |
| T1 | 4552 (69) | 1 | 1 | Reference | 1 | 1 | Reference | 1 | 1 | Reference |  |
| T2 | 1510 (24) | 0.70 | (0.44,1.12) | 0.139 | 0.79 | (0.49,1.27) | 0.335 | 0.80 | (0.50,1.28) | 0.347 |  |
| T3 | 2788 (46) | 0.67 | (0.46,0.98) | 0.040 | 0.83 | (0.56,1.23) | 0.353 | 0.87 | (0.59,1.29) | 0.495 |  |
| Per+SD |  | 0.76 | (0.64,0.91) | 0.003 | 0.85 | (0.71,1.02) | 0.075 | 0.87 | (0.72,1.04) | 0.124 |  |
| **DII** |  |  |  |  |  |  |  |  |  |  |  |
| T1 | 2950 (33) | 1 | 1 | Reference | 1 | 1 | Reference | 1 | 1 | Reference |  |
| T2 | 2950 (46) | 1.59 | (0.99,2.53) | 0.054 | 1.38 | (0.86,2.21) | 0.187 | 1.35 | (0.84,2.17) | 0.216 |  |
| T3 | 2950 (60) | 1.90 | (1.14,3.16) | 0.013 | 1.51 | (0.90,2.54) | 0.120 | 1.47 | (0.87,2.47) | 0.150 |  |
| Per+SD |  | 1.38 | (1.11,1.72) | 0.004 | 1.23 | (0.98,1.54) | 0.077 | 1.21 | (0.96,1.51) | 0.106 |  |

**Note:**

Model 1: age (continuous), ethnicity/race (Mexican American, Other Hispanic, Non-Hispanic White, Non-Hispanic Black, Other Race - Including Multi-Racial), total energy intake (continuous).

Model 2: age (continuous), ethnicity/race (Mexican American, Other Hispanic, Non-Hispanic White, Non-Hispanic Black, Other Race - Including Multi-Racial), educational level (less than high school, high school and above), ratio of family income to poverty (< 1.3, 1.3-3.5, ≥ 3.5), smoking status (Yes or No), alcohol consumption (continuous), physical activity (adequate, inadequate), total energy intake (continuous).

Model 3: age (continuous), ethnicity/race (Mexican American, Other Hispanic, Non-Hispanic White, Non-Hispanic Black, Other Race - Including Multi-Racial), educational level (less than high school, high school and above), ratio of family income to poverty (< 1.3, 1.3-3.5, ≥ 3.5), smoking status (Yes or No), alcohol consumption (continuous), physical activity (adequate, inadequate), BMI (continuous), history of diabetes (Yes or No), total energy intake (continuous).

1. values less than 0.05 (p < 0.05) were considered significant.

**Abbreviations:** CVD=cardiovascular disease, CKM=Cardiovascular-Kidney-Metabolic Syndrome, NHANES=National Health and Nutrition Examination Survey, T=tertile, SD=standard deviation, BMI=body mass index, AMED=Alternate Mediterranean Diet, DII=Dietary Inflammation Index, HR=hazard ratio, CI=confidence interval, N=number.
